# Unveiling the microbiome of hydroponically cultivated lettuce: impact of *Phytophthora cryptogea* infection on plant-associated microorganisms

**DOI:** 10.1093/femsec/fiae010

**Published:** 2024-02-05

**Authors:** Liese Vlasselaer, Sam Crauwels, Bart Lievens, Barbara De Coninck

**Affiliations:** Plant Health and Protection Laboratory, Division of Crop Biotechnics, Department of Biosystems, KU Leuven, Willem de Croylaan 42, B-3001 Leuven, Belgium; KU Leuven Plant Institute, Kasteelpark Arenberg 31, B-3001 Leuven, Belgium; KU Leuven Plant Institute, Kasteelpark Arenberg 31, B-3001 Leuven, Belgium; Laboratory for Process Microbial Ecology and Bioinspirational Management, Center of Microbial and Plant Genetics, Department of Microbial and Molecular Systems, KU Leuven, Willem de Croylaan 46, B-3001 Leuven, Belgium; KU Leuven Plant Institute, Kasteelpark Arenberg 31, B-3001 Leuven, Belgium; Laboratory for Process Microbial Ecology and Bioinspirational Management, Center of Microbial and Plant Genetics, Department of Microbial and Molecular Systems, KU Leuven, Willem de Croylaan 46, B-3001 Leuven, Belgium; Plant Health and Protection Laboratory, Division of Crop Biotechnics, Department of Biosystems, KU Leuven, Willem de Croylaan 42, B-3001 Leuven, Belgium; KU Leuven Plant Institute, Kasteelpark Arenberg 31, B-3001 Leuven, Belgium

**Keywords:** bacterial community composition, endosphere, hydroponics, microbiome diversity, pathogen, rhizosphere

## Abstract

Understanding the complex interactions between plants and their associated microorganisms is crucial for optimizing plant health and productivity. While microbiomes of soil-bound cultivated crops are extensively studied, microbiomes of hydroponically cultivated crops have received limited attention. To address this knowledge gap, we investigated the rhizosphere and root endosphere of hydroponically cultivated lettuce. Additionally, we sought to explore the potential impact of the oomycete pathogen *Phytophthora cryptogea* on these microbiomes. Root samples were collected from symptomatic and nonsymptomatic plants in three different greenhouses. Amplicon sequencing of the bacterial 16S rRNA gene revealed significant alterations in the bacterial community upon *P. cryptogea* infection, particularly in the rhizosphere. Permutational multivariate analysis of variance (perMANOVA) revealed significant differences in microbial communities between plants from the three greenhouses, and between symptomatic and nonsymptomatic plants. Further analysis uncovered differentially abundant zero-radius operational taxonomic units (zOTUs) between symptomatic and nonsymptomatic plants. Interestingly, members of *Pseudomonas* and *Flavobacterium* were positively associated with symptomatic plants. Overall, this study provides valuable insights into the microbiome of hydroponically cultivated plants and highlights the influence of pathogen invasion on plant-associated microbial communities. Further research is required to elucidate the potential role of *Pseudomonas* and *Flavobacterium* spp. in controlling *P. cryptogea* infections within hydroponically cultivated lettuce greenhouses.

## Teaser

Impact of *Phytophthora cryptogea* on the rhizosphere and root endosphere microbiome of hydroponically cultivated lettuce.

## Introduction

In recent years, there has been a growing recognition of the crucial role played by plant-associated microorganisms in enhancing plant health and productivity, especially in the context of sustainable crop production (Berg et al. 2014a,b). Plant-associated microorganisms can contribute to disease suppression either directly by antagonizing the pathogen, or indirectly by priming the plant’s immune system (also known as induced resistance; IR) (Berendsen et al. 2012, Raaijmakers and Mazzola 2012, Zamioudis and Pieterse 2012, Köhl et al. 2019, De Kesel et al. 2021). In this regard, a growing body of research is highlighting the impact of the root microbiome in promoting plant health. Specifically, the root rhizosphere, defined as the narrow region of soil (or substrate) surrounding the roots plays a vital role, as it is harboring a diverse range of microorganisms, including bacteria, fungi, oomycetes, archaea, protozoa, and algae. These microorganisms are attracted by the plant through root exudates giving rise to complex microbial root communities that may suppress soil-borne pathogens or provide other benefits to the plant (Costa et al. 2007, Hartmann et al. 2008, Badri and Vivanco 2009, Lahlali et al. 2022). Although much research has focused on the rhizosphere, it is important to note that microorganisms can colonize other parts of the plant as well, which may also contribute to plant growth and plant health. The root endosphere, for instance, which encompasses all microorganisms residing within the plant roots, is increasingly recognized for its unique interaction with the plant and its role in promoting plant health (Ryan et al. 2008, Reinhold-Hurek and Hurek 2011). Moreover, in addition to microbial individuals both direct and indirect interactions between microorganisms can have significant impacts on plant health. Therefore, understanding the overall microbiome, its functioning and its interactions with the plant and environment is crucial to promote plant health (Berendsen et al. 2012).

In addition to the beneficial microorganisms that promote plant health and growth, the microbial communities associated with plants also include pathogens that can infect plants and alter the plant microbiome. For example, when plants are infected with pathogens, the signaling of defense-related hormones, such as salicylic acid and jasmonic acid, can change, leading to altered root exudates and, in turn, to changes in microbial community composition (Badri et al. 2008, Pieterse et al. 2009, Berendsen et al. 2012). Studies have also shown that substantial differences occur in the microbiomes of naturally infected plants and their healthy counterparts originating from the same field, indicating that pathogens can alter the plant microbiome (Suhaimi et al. 2017, Wei et al. 2018, Shi et al. 2021). Also in the case of artificial pathogen inoculation, significant differences in microbial communities were found following pathogen infection (Chapelle et al. 2016, Snelders et al. 2020). However, results seem to be context-dependent, and show either a higher relative abundance of plant-beneficial microbes in noninfected plants (Suhaimi et al. 2017, Wei et al. 2018) or an increase in their relative abundance in infected plants (Chapelle et al. 2016). The latter supports the “cry-for-help” strategy, proposing that plants recruit beneficial microorganisms when exposed to stress, which can result in diminished disease severity (Bakker et al. 2018). For example, in sugar beet plants artificially infected by the soilborne fungal pathogen *Rhizoctonia solani*, a higher relative abundance of bacterial families which are known for their antifungal potential was observed compared to noninfected plants (Chapelle et al. 2016). By contrast, a significantly lower relative abundance of plant-beneficial microbes was found in *Ralstonia solanacearum*-infected tomato plants compared to the noninfected plants (Wei et al. 2018). Similarly, higher relative abundance of the plant beneficials *Pseudomonas* spp. and *Bacillus* spp. was found in healthy banana endospheres compared to the endosphere of bacterial-wilt infected banana plants (Suhaimi et al. 2017). To date, most attention has been given to microbiome responses to fungal and bacterial pathogens. By contrast, only little is known on how microbial communities respond to oomycete plant pathogens such as *Phytophthora* spp. and *Pythium* spp., despite the fact that this group represents one of the most economically important and widespread categories of plant pathogens (Sullam and Musa 2021). In a recent study by Gómez-Pérez et al. (2023), it has been demonstrated that the oomycete pathogen *Albugo candida* releases proteins into the host plant apoplast repressing plant-associated bacteria. The antimicrobial activity of these proteins was found to enhance host colonization by the pathogen (Gómez-Pérez et al. 2023, Rovenich and Thomma 2023).

Although plant microbiomes have received extensive attention in recent times, primarily focusing on soil-cultivated crops, there is still limited information available on the microbiomes of hydroponically cultivated crops and how they are affected by plant pathogens. To the best of our knowledge, so far only one study has been conducted on the influence of a pathogen on the microbiome of hydroponically grown plants. This study demonstrated that the root microbiome of tomato plants significantly differs between healthy plants and plants infected with rhizogenic agrobacteria (Vargas et al. 2022). The root microbiome of healthy plants showed a higher relative abundance of *Paenibacillus* spp. (Vargas et al. 2022), from which specific lineages have antagonistic activity against rhizogenic agrobacteria (Bosmans et al. 2017). Nevertheless, further studies are needed to increase our understanding of the microbiomes of hydroponically cultivated crops.

The main goal of this study was to investigate the impact of oomycete plant pathogens on microbial communities associated with hydroponically grown crops, focusing on both rhizosphere and endosphere bacterial communities. More specifically, we focused on the interaction between hydroponic lettuce (*Lactuca sativa* L.) and *Phytophthora cryptogea* as our study system. Currently, lettuce is widely grown in hydroponic systems as a rapidly growing leafy vegetable (Safaei et al. 2015), whereas in the past, lettuce was predominantly cultivated in soil-bound systems. The choice of the cultivation method is largely determined by the cultivar, environmental conditions, potential disease pressure, and the capital of the grower (Parkell et al. 2015). In soil-bound cultivation, lettuce is often grown in an intensive monocropping system, through which soil-borne diseases are likely to build up. In Flanders, a switch toward hydroponic lettuce cultivation has been implemented due to the high disease pressure caused by soil-borne pathogens such as *Fusarium oxysporum* f. sp. *lactucae*, coupled with the phasing out of soil fumigation chemicals (e.g. methyl bromide) (Vandevelde 2019, Claerbout 2020). Other major advantages of hydroponics over soil-bound cultivation are shorter production times, reduced labor requirements as some agricultural practices, such as weeding, tilling, and spraying can be eliminated, more efficient utilization of available space, and reduced water usage through the recycling of nutrient solutions. The major disadvantage, however, is the easy spread of water-borne diseases through the recirculation system (Sharma et al. 2018, Vandevelde 2019). From 2017 onward, the devastating root rot-causing oomycete *P. cryptogea* is severely threatening hydroponic cultivation of lettuce. Notably, it was found that *P. cryptogea* tends to prevail during warm periods (e.g. heatwaves), which will most probably become worse in the future due to climate change (Berckmoes and Van Cleemput 2021). The economic repercussions of this pathogen were estimated to lead to economic losses up to €50 000/ha/year (I. Vandevelde, personal communication). *Phytophthora cryptogea* produces flagellated, asexual zoospores, which can attach to the root surface and germinate to form a germination tube or appressorium on the host tissue, after which hyphae invade the plant tissue (Hubrechts and De Marez 2014, Pettitt 2015, Berckmoes and Van Cleemput 2021). First, a slimy coat around the roots is formed, after which the roots start to show rotting symptoms followed by complete rotting of the lettuce crop (Figure S1, Supporting Information). Because of the motile zoospores, which can easily spread through the recirculated nutrient solution, contamination of the entire system can occur rapidly (Hubrechts and De Marez 2014, Pettitt 2015, Sharma et al. 2018), leading to major economic losses. To date, there are no plant protection products available to control *P. cryptogea* in hydroponically cultivated lettuce.

The aim of this study was to investigate the rhizosphere and root endosphere of hydroponically cultivated lettuce. Additionally, we sought to explore the hypothesis that *P. cryptogea* affects the bacterial communities of the lettuce rhizosphere and root endosphere. To test this hypothesis, both symptomatic and nonsymptomatic plants were sampled from three greenhouses naturally infested with *P. cryptogea*. Bacterial communities were examined by deep sequencing of partial 16S ribosomal RNA (rRNA) gene amplicons, and differences between healthy and symptomatic plants were described. By studying naturally infested greenhouses, we aimed to gain a better understanding of the impact of this important pathogen on plant-associated microbial communities under real-world conditions.

## Material and methods

### Sampling and DNA extraction

Our study was done in collaboration with three commercial hydroponic lettuce growers in Flanders (Sint-Katelijne-Waver, Belgium), referred to as greenhouse 1, 2, and 3. In all greenhouses, lettuce was cultivated in a nutrient film technique system, more particularly a mobile gutter system, in which plant density can be adapted according to the plant growth stage. Climatic conditions, EC, and pH of the nutrient solution were similar for each greenhouse. The plants in each greenhouse were sourced from the same plant nursery and were grown in substrate cubes containing 85% black peat and 15% wood fiber. Greenhouses 1 and 3 were cultivating butterhead lettuce, cv. Emeldia (Rijk Zwaan) and cv. Finley (Enza Zaden), respectively, while greenhouse 2 was cultivating multicolor lettuce, consisting of cv. Lugano, Satine, and Xodos (Rijk Zwaan). In each greenhouse, symptomatic and nonsymptomatic plants were collected including their substrate cubes, which were selected based on the visual presence or absence of typical *P. cryptogea* symptoms (i.e. brown or necrotic roots). Sampling was conducted without a predefined time point, as diseased plants were not always available. Instead, samples were taken when *P. cryptogea* infection was observed (i.e. during warmer periods in the growth season). To confirm *P. cryptogea* as the causing agent of the symptoms, a qPCR assay was performed (see below), and for some plants the pathogen was isolated as described previously (Drenth and Sendall 2001). Plants were sampled after 21 (greenhouses 2 and 3) or 28 days (greenhouse 1) of growth on the gutters in June 2021 (greenhouses 2 and 3) and August 2021 (greenhouse 1), meaning that plants were almost fully grown. In general, lettuce has a cycle length of 21–35 days in summer (depending on the variety). On average in the investigated greenhouses, 220 plants/m^2^/year were cultivated during 8–12 cycles/year. To avoid effects of age, both symptomatic and nonsymptomatic plants were sampled at the same time in the same and/or adjacent gutters.

Following cooled transport of the plants to the laboratory, samples were processed within 24 h after sampling based on the protocols described by Lakshmanan et al. (2017) and Bergna et al. (2018) (Figure S2, Supporting Information). Briefly, all roots outside the substrate cube of a plant were collected, as these roots came first into contact with the pathogen, and cut into 2 cm pieces. Subsequently, 20 ml of sterile phosphate buffered saline (PBS) was added to 2 g of randomly pooled root pieces. Next, root pieces were vortexed for 30 s and rinsed with another 10 ml of PBS, followed by sonication at 14.5 kHz for 30 s in 15 ml of PBS for removal of remaining external microbes. Finally, roots were rinsed again with 5 ml of PBS. After each step, the PBS solution was collected and pooled per plant. The pooled solution was then centrifuged at 4°C at 5000 × *g* for 15 min. Next, the supernatant was discarded and the pellet was dissolved in 1 ml sterile 0.9% NaCl. The samples collected in this way represented microbiome samples of the rhizosphere. The corresponding roots were further processed for sampling of the endosphere microbiome. Therefore, the roots were rinsed with 25 ml of sterile demineralized water, followed by surface sterilization with 3% bleach for 5 min and three times rinsing with 25 ml sterile demineralized water. Surface-sterilized roots were then used for further processing. A total of eight samples from nonsymptomatic plants and eight samples from symptomatic plants were analyzed for both greenhouses 1 and 2. Additionally, seven samples from nonsymptomatic plants and seven samples from symptomatic plants were analyzed for greenhouse 3. For all greenhouse, both the rhizosphere and endosphere were analyzed (Table S1, Supporting Information). From each sample, genomic DNA was extracted using the DNeasy PowerSoil Pro Kit (Qiagen, Hilden, Germany) following the manufacturer’s instructions with one modification: in the second step the use of a vortex adapter was replaced by two cycles of 30 s (with a 10 s break in between) in the Precellys^®^24 Tissue Homogenizer at a speed of 6000 r/m. To this end, 500 µl of the cell pellet was used for rhizosphere samples and ∼250 mg of the roots for endosphere samples. A negative control in which the sample material was replaced by sterile, DNA-free water was included to confirm absence of reagent contamination.

### Microbiome analysis

For microbiome analysis, all DNA samples were subjected to PCR amplification of the bacterial hypervariable V4 region of the 16S rRNA gene using the Illumina barcoded primer pair 515F/806R (Caporaso et al. 2011), designed according to Kozich et al. (2013) (dual-index sequencing strategy; Table S1, Supporting Information). A negative control for PCR amplification, in which sterile, DNA-free water was used instead of the DNA template, was included in each PCR run. Additionally, a DNA sample from a mock community composed of diverse bacteria was included as a reference (Table S2, Supporting information). PCRs were performed in a 40 µl reaction volume, containing 1x Titanium Taq PCR Buffer (Takara Bio), 0.15 mM of each dNTP (Invitrogen^TM^), 1x Titanium Taq DNA Polymerase (Takara Bio), 0.5 µM of each primer, and 2 µl DNA template. Amplification started with an initial denaturation for 2 min at 94°C, followed by 34 cycles of denaturation, annealing and extension for 45 s each at 94°C, 59°C, and 72°C, respectively, and ended with a final elongation for 10 min at 72°C. Next, amplicons were purified using Agencourt AMPure XP magnetic beads (Beckman Coulter Genomics GmbH, South Plainfield, UK) following the manufacturer’s instructions. The concentration of purified DNA fragments was then measured using a Qubit high sensitivity fluorometer (Invitrogen^TM^, Carlsbad, USA) and diluted to a concentration of 20 nM. Next, samples were pooled into a library and subjected to ethanol precipitation, after which the library was loaded on a 1.5% agarose gel. Subsequently, the target band representing fragments of around 400 bp was excised from the gel and purified using the QIAquick Gel Extraction Kit (Qiagen). Finally, the DNA library was diluted to 2 nM and sent for sequencing at the Center for Medical Genetics (University of Antwerp, Antwerp, Belgium), using an Illumina MiSeq sequencer with a v2 500-cycle reagent kit (Illumina, San Diego, USA).

Sequences were received in the form of a demultiplexed FASTQ file with removed barcodes and primer sequences. To merge paired-end reads, USEARCH (v11.0.667) was used to form consensus sequences (Edgar 2013), allowing for no more than 10 mismatches in the overlap region. The resulting sequences were trimmed at the 250th base, and any reads with a length shorter than 250 bp or a total expected error threshold above 0.1 were discarded using USEARCH (v11.0.667). Next, Mothur (v1.39.5) was used with the SILVA database (v1.38) to identify and remove mitochondrial, chloroplast, or other nontarget sequences using the commands “classify.seqs” and “remove.lineage” or “get.lineage”. Bacterial sequences were classified into zero-radius operational taxonomic units [zOTUs, also known as amplicon sequence variants (ASVs)] (Edgar 2016, Callahan et al. 2017) using the UNOISE3 algorithm implemented in USEARCH (Edgar and Flyvbjerg 2015). The microDecon (v1.2.0) package in R (v3.5.2) was used to correct the data set for potential contaminants based on zOTU prevalence in the samples compared to the mean of the PCR negative control samples (Davis et al. 2018, R Core Team 2018). The DNA extraction control was removed from the data set as it yielded low sequence numbers and no additional zOTUs compared to the PCR controls. To further eliminate potential contaminants, zOTUs with a relative abundance below 0.1% per sample were removed from the data set (Gloder et al. 2021). In this way, all members of the mock community were detected, while contaminating reads were discarded (Table S3, Supporting Information), indicating that the experimental conditions were met to obtain robust data. Finally, in order to correct for uneven sequencing depth among samples, samples were rarefied to an equal number of sequences. Specifically, endosphere samples were rarefied to 750 reads, while rhizosphere samples were rarified to 25 000 reads. Samples that did not yield enough sequence reads were discarded from further data analysis (12 samples in total). For the rhizosphere, two samples from symptomatic plants of greenhouse 1 and one sample from symptomatic plants of greenhouses 2 and 3 were discarded. For the endosphere, one sample from nonsymptomatic plants of greenhouse 1 and six samples of nonsymptomatic plants of greenhouse 2 were discarded, as well as one sample from symptomatic plans of greenhouse 2. The taxonomic origin of each zOTU was determined with the SINTAX algorithm as implemented in USEARCH based on the SILVA Living Tree Project v123. Further, the identity of the most important zOTUs was verified with a BLAST search in GenBank against type materials. The BLAST search was extended to the entire database when no significant similarity was found with type materials (< 97% identity). Sequences obtained in this study were deposited in the Sequence Read Archive (SRA) at NCBI under Bioproject PRJNA947809.

### Quantification of *P. cryptogea* and bacterial densities

Prior to subjecting the samples to microbiome analysis, *P. cryptogea* was quantified in all samples investigated using a probe-based qPCR assay with the primers Pcry-F (5′-TGACGTTGCTGGTTGTGGAGG-3′) and Pcry-R (5′-GACACCCTACTTCGCACCACA-3′) and the FAM-labeled, double quenched probe Pcry-P (5′-/56-FAM/ATTAAACGC/ZEN/CGCAGCAGACAAACC/3IABkFQ/-3′). These primers amplified a section of the internal transcribed spacer (ITS) region of *P. cryptogea*. The qPCR assay was performed in a CFX96 Touch Real-Time PCR Detection System (Bio-Rad Laboratories, Inc.) in a 20 µl reaction volume, containing 1x PrimeTime Gene Expression Master Mix (Integrated DNA Technologies, Inc.), 0.25 nM of each primer, 0.25 µM of the probe, and 2 µl DNA template (10 ng/µl). The qPCR assay was initiated with an initial denaturation for 3 min at 95°C, followed by 40 cycles of denaturation and annealing/extension for 5 s and 30 s at 95°C and 60°C, respectively, according to the manufacturer’s instructions. As a negative control, DNA-free water was used instead of DNA template. C_T_ values lower than 40 were considered to be positive, which was consistently lower than C_T_ values obtained for blank samples. Evaluation of the specificity of the assay against various fungi and oomycetes, including the target species as well as a number of close relatives, revealed that the assay was highly specific under these conditions. Quantification of *P. cryptogea* DNA was performed based on a standard curve generated with a 10-fold dilution series of ITS amplicons from *P. cryptogea* (E = 91.1%; *R*^2^ = 0.999).

In the rhizosphere samples, also the bacterial density was assessed through a SYBR Green-based qPCR assay using unmodified 515F/806R primers to determine the bacterial 16S rRNA gene copy numbers (for details, see Borremans et al. 2019). The qPCR assay was performed in a CFX96 Touch Real-Time PCR Detection System (Bio-Rad Laboratories, Inc.) in a 20 µl reaction volume, containing 1x SsoAdvanced universal SYBR® Green supermix (Bio-Rad Laboratories, Inc.), 0.20 µM of each primer and 2 µl DNA template (10 ng/µl). DNA concentrations were determined based on a standard curve established from the analysis of a 10-fold dilution series of 16S rRNA gene amplicons from *Bacillus amyloliquefaciens* QST713 (E = 87.24%; *R*^2^ = 0.999). A negative control in which template DNA was replaced by sterile, DNA free water was included in every qPCR run performed, and a C_T_ value of 40 was taken as the detection threshold, which was below the C_T_ value obtained for any negative control. Given that the 16S rRNA gene primers used may also amplify mitochondrial and chloroplast DNA in plants (King et al. 2012; Table S4, Supporting Information), it was not possible to determine the bacterial density in the endosphere samples.

### Statistical analyses

Statistical analyses were performed using Rstudio 2022.02.2+485 (R Core Team 2022). As the number of rarefied reads for rhizosphere and endosphere samples was too different, the data set was split in two subsets, representing either rhizosphere or endosphere samples, and were analyzed separately. To determine whether the sequencing depth was sufficient to estimate microbial diversity, rarefaction curves were made using the *vegan* package in R, plotting the number of observed zOTUs in function of the number of sequences. Next, the alpha diversity metrics observed zOTU richness (i.e. the number of zOTUs present in a sample) and the Simpson’s diversity index, taking into account the number of zOTUs present as well as the relative abundance of each zOTU, were calculated. The alpha diversity metrics and qPCR data were statistically analyzed using a Wilcoxon (Mann–Whitney U) test, which can be used for small and unequal sample sizes (Smalheiser 2017, Zhu 2021). Further, based on the Hellinger transformed relative abundance data, the bacterial community composition (beta diversity) was visualized by nonmetric multidimensional scaling (NMDS) using the Bray–Curtis coefficient as distance measure in the R software package *vegan*. To test the hypothesis that infection with *P. cryptogea* altered the bacterial community composition, i.e. that there were significant differences in bacterial communities between samples from symptomatic and nonsymptomatic plants, a permutational multivariate analysis of variance (perMANOVA) was performed on the same data set. In order to identify zOTUs that were differentially abundant in samples from nonsymptomatic plants compared to symptomatic plants a combination of three methods was used. These included linear discriminant analysis effect size (LEfSe), DESEq2, and EdgeR. Although the latter two methods are commonly used to analyze differential gene expression with RNA-seq data, they are also increasingly used in microbiome studies to identify zOTUs that are differentially abundant among conditions (Halfvarson et al. 2017, Jiang et al. 2022). Analyses were carried out using the *phyloseq* package, followed by the functions *run_lefse, run_deseq2* and *run_edger* (*microbiomeMarker* package). Differentially abundant zOTUs were presented using volcano plots, with (1) a cutoff of 4 for the fold change, (2) a linear discriminant analysis score cutoff of 2 for LEfSe analysis, and (3) an adjusted *P*-value of .05.

## Results

**Figure 1.**
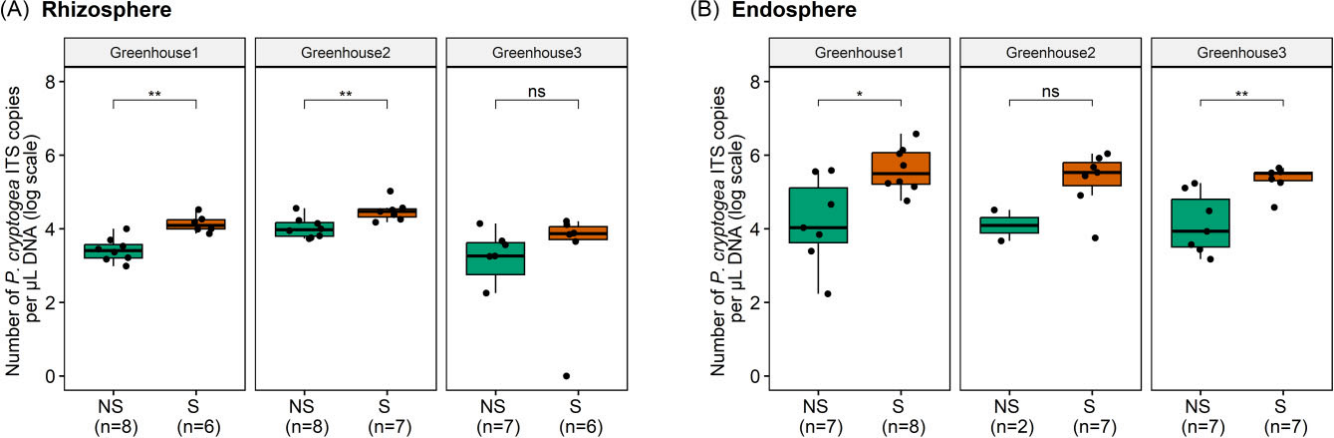
Number of *P. cryptogea* ITS copies per µl DNA (log scale) present in roots of nonsymptomatic (NS) and symptomatic (S) plants, for both the rhizosphere (A) and root endosphere (B). Results are presented for the three greenhouses separately. The lower, middle and upper lines of the boxplots correspond to the first quartile, median and third quartile, respectively, while the whiskers represent the range from the minimum to the maximum. Data points represent the different replicates (number provided between brackets). Significant differences between symptomatic and nonsymptomatic plants are shown by one or more asterisks (*P* > .05 (ns), *P* ≤ .05 (*), *P* ≤ .01 (**), and *P* ≤ .001 (***)).

In order to assess whether bacterial communities in the rhizosphere and root endosphere of hydroponically grown lettuce plants differ between nonsymptomatic and symptomatic plants, both nonsymptomatic and symptomatic lettuce plants were simultaneously collected from different greenhouses infested with *P. cryptogea*. First, using a qPCR assay targeting *P. cryptogea* DNA, we confirmed that plants that were classified as symptomatic plants, based on visible symptoms, were infected with *P. cryptogea*. Generally, a significantly higher number of *P. cryptogea* ITS copies was detected in samples of symptomatic plants compared to the nonsymptomatic plants for both the rhizosphere (Fig. 1A) and the endosphere (Fig. 1B) in each greenhouse, except for the rhizosphere samples of greenhouse 3 and the endosphere samples of greenhouse 2. However, also in most nonsymptomatic plants very low amounts of *P. cryptogea* DNA were detected.

**Figure 2.**
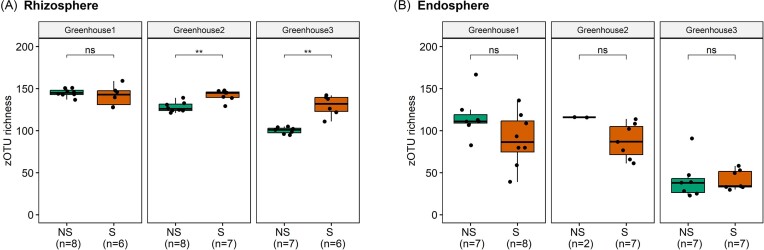
zOTU of bacterial communities of the rhizosphere (A) and root endosphere (B) of lettuce plants collected at three hydroponic lettuce greenhouses for both nonsymptomatic (NS) and symptomatic (S) plants. Results are presented for the three greenhouses separately. The lower, middle and upper lines of the boxplots correspond to the first quartile, median and third quartile, respectively, while the whiskers represent the range from the minimum to the maximum. Data points represent the different replicates (number provided between brackets). Significant differences between symptomatic and nonsymptomatic plants are indicated by one or more asterisks (*P* > .05 (ns), *P* ≤ .05 (*), and *P* ≤ .01 (**)).

Next, DNA samples were subjected to a microbial community analysis. In total, 2 405 622 reads were obtained for the rhizosphere samples, while 2 712 629 reads were retrieved for the endosphere samples. After quality control, the number of obtained reads was reduced to 2 267 428 and 1 056 933, respectively, particularly due to the presence of chloroplast and mitochondrial DNA sequences in the endosphere samples (Table S4, Supporting Information). After decontamination and rarefying of the data, sequence analysis revealed a total of 633 bacterial zOTUs for the rhizosphere data set and 734 zOTUs for the endosphere data set. Rarefaction curves reached saturation (especially for rhizosphere samples) or tended to approach saturation (Figure S3, Supporting Information), indicating that the sequencing depths were sufficient to cover microbial diversity. With regard to alpha diversity, the number of zOTUs per sample ranged from 95 to 159 for the rhizosphere samples and from 23 to 167 zOTUs for the endosphere samples (Fig. 2A and B). For each greenhouse, a significantly higher zOTU richness was found in the rhizosphere samples compared to the endosphere samples (Figure S4, Supporting Information). With regard to the rhizosphere samples, a significantly higher richness was found in the samples of symptomatic compared to the nonsymptomatic plants for the plants from greenhouses 2 and 3, while no difference was found for greenhouse 1 (Fig. 2A). However, for the endosphere samples, no significant difference in zOTU richness was found between the symptomatic and nonsymptomatic plants for none of the greenhouses (Fig. 2B). In terms of the Simpson Diversity Index, no significant differences were found between symptomatic and nonsymptomatic plants for both the rhizosphere and endosphere, except for a significantly higher Simpson Diversity Index for the rhizosphere samples of symptomatic compared to the nonsymptomatic plants from greenhouse 3 (Figure S5A and B, Supporting Information).

**Figure 3.**
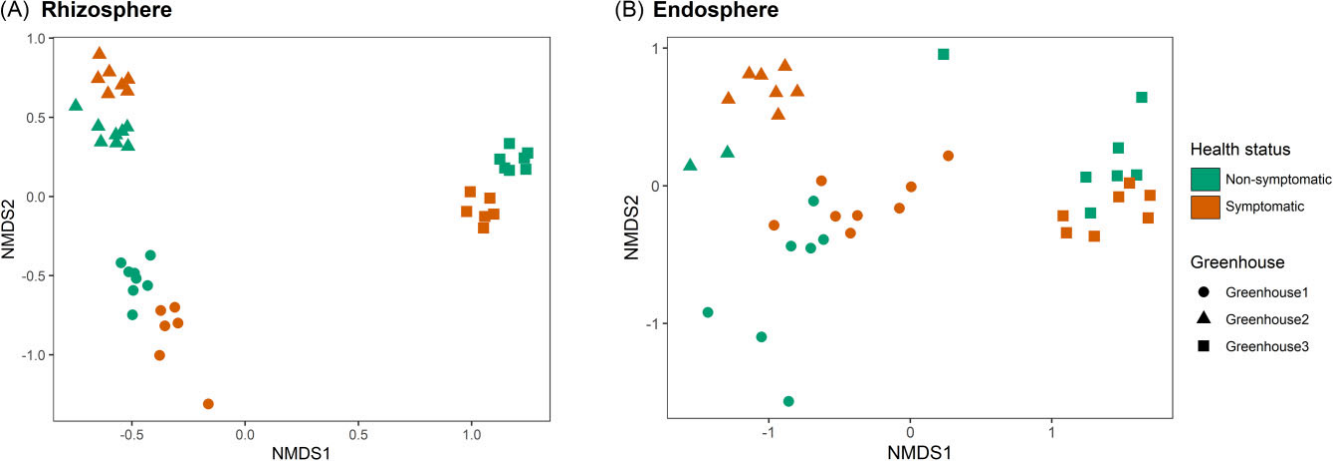
NMDS ordination plots based on relative abundance data of the bacterial communities in the rhizosphere (stress = 0.07) (A) and endosphere (stress = 0.11) (B) of lettuce root samples taken at three hydroponic lettuce greenhouses. Samples were taken from both nonsymptomatic and symptomatic plants. The closer two data points, the more similar the bacterial communities are.

**Figure 4.**
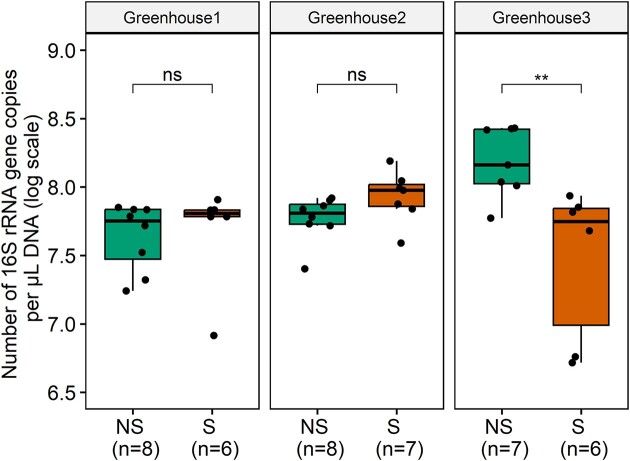
Number of 16S rRNA gene copies per µl DNA (log scale) present in roots of nonsymptomatic (NS) and symptomatic (S) lettuce plants, collected at three hydroponic lettuce greenhouses, for the rhizosphere. Results are presented for the three greenhouses separately. The lower, middle and upper lines of the boxplots correspond to the first quartile, median and third quartile, respectively, while the whiskers represent the range from the minimum to the maximum. Data points represent the different replicates (number provided between brackets). Significant differences are indicated with one or more asterisks (*P* > .05 (ns), *P* ≤ .05 (*), *P* ≤ .01 (**), and *P* ≤ .001 (***)).

NMDS plots for rhizosphere (stress = 0.07) and endosphere samples (stress = 0.11) showed a clear clustering between samples from the different greenhouses, especially for the rhizosphere samples (Fig. 3A). With regard to the rhizosphere, samples from greenhouse 3 were separated from the other greenhouses by NMDS1, while NMDS2 separated samples from greenhouse 1 and greenhouse 2. Additionally, NMDS2 clearly separated samples from nonsymptomatic and symptomatic plants for each greenhouse. Similar observations can be made for the endosphere samples, but separation here is less pronounced (Fig. 3B). PerMANOVA revealed significant differences in bacterial community composition between plants of the three lettuce greenhouses (rhizosphere: *F* = 36.16, *P* < .001 and endosphere: *F* = 13.16, *P* < .001) and across health status (rhizosphere: *F* = 3.70, *P* < .01 and endosphere: *F* = 1.98, *P* < .05). To estimate the absolute abundance of bacterial cells, a qPCR assay was performed to determine the number of 16S rRNA gene copies (Fig. 4). Generally, no significant differences in gene copy numbers were found between rhizosphere samples from symptomatic and nonsymptomatic plants (Fig. 4). However for greenhouse 3, a significantly higher number of 16S rRNA gene copies was observed in the nonsymptomatic plants for the rhizosphere, although a lower alpha diversity was observed (Figs 2 and 4).

**Figure 5.**
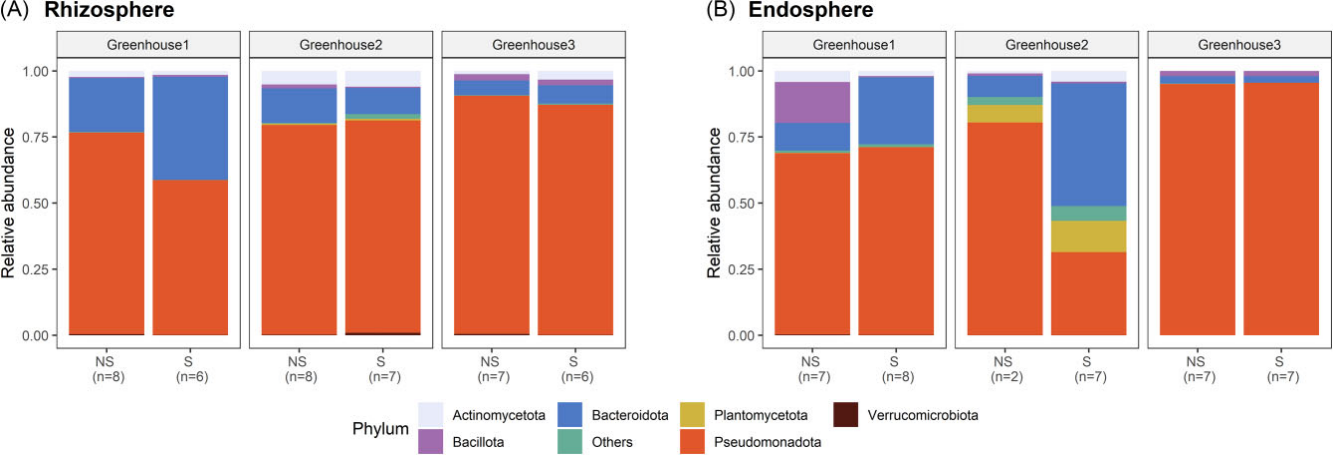
Phylum composition in the rhizosphere (A) and endosphere (B) of nonsymptomatic (NS) and symptomatic (S) lettuce plants collected at three hydroponic lettuce greenhouses. Results are presented for the three greenhouses separately. The number of replicates included is given between brackets.

When zooming in at the bacterial community composition, a high variability can be observed between the three greenhouses. Overall, the bacterial community is dominated by the phylum of Pseudomonadota (Proteobacteria) in both rhizosphere and endosphere samples and both nonsymptomatic and symptomatic plants for all greenhouses (Fig. 5A and B). Other abundant phyla in the rhizosphere, for both symptomatic and nonsymptomatic plants, are Bacteroidota (Bacteroidetes), Actinomycetota (Actinobacteria), Bacillota (Firmicutes), and Verrucomicrobiota (Verrucomicrobia) (Fig. 5A). However, at greenhouse level, a significantly higher relative abundance of Bacteroidota was found in the symptomatic compared to the nonsymptomatic plants of greenhouse 1 (Fig. 5A; Figure S6, Supporting Information), while a significantly higher relative abundance of Pseudomonadota and Verrucomicrobiota was observed in the nonsymptomatic compared to the symptomatic plants. For greenhouse 2, a significantly higher relative abundance of Bacteroidota and Bacillota and a significantly lower relative abundance of Verrucomicrobiota was observed in the nonsymptomatic compared to the symptomatic plants. For greenhouse 3, a significantly higher relative abundance of Actinomycetota was found in the symptomatic compared to the nonsymptomatic plants, while a significantly lower relative abundance of Pseudomonadota and Verrucomicrobiota was found in these plants. Regarding the endosphere, the bacterial communities were also dominated by Pseudomonadota, Bacteroidota, Bacillota, and Actinomycetota, although more variability was present between the greenhouses (Fig. 5B). In greenhouse 2, one of the most abundant phyla was Planctomycetota (Planctomycetes), which was mainly absent in greenhouses 1 and 3. Significant differences between symptomatic and nonsymptomatic plants at greenhouse level were only found in greenhouse 1, where a significantly higher relative abundance of Bacteroidota was found in the symptomatic compared to the nonsymptomatic plants.

**Figure 6.**
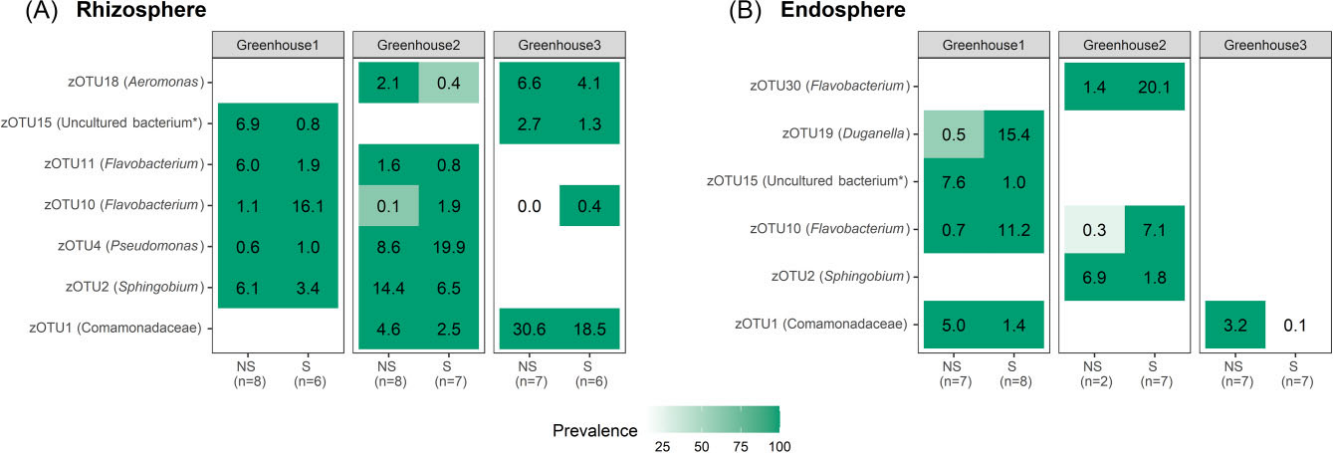
Summary of the relative abundance and prevalence of the zOTUs, which are differentially abundant between nonsymptomatic (NS) and symptomatic (S) plants according to the combined results of the DESeq2, EdgeR, and LEfSe analyses and with an overall relative abundance of ≥ 5% for at least one greenhouse and symptomatic/nonsymptomatic plants. Results are presented for the three greenhouses separately for both the rhizosphere (A) and the endosphere (B). For each zOTU, the relative abundance is given as a number (%), while the color represents prevalence (i.e. fraction of samples in which the zOTU was present; white is absent). The number of replicates included is given between brackets. zOTUs are identified by a BLAST search against type materials in GenBank. When no significant similarity was found with type materials (< 97%), the BLAST analysis was performed against the entire GenBank (indicated with an asterisk). Identifications were performed at genus level; when identical scores were obtained for different genera, identifications were performed at family level.

To identify bacterial zOTUs linked to nonsymptomatic and/or symptomatic plants, a differential abundancy analysis was performed using DESeq2, EdgeR, and LEfSe analysis (Figures S7–S9, Supporting Information). The differential abundant zOTUs shared between the three analysis methods and with an overall relative abundance larger than 5% for at least one greenhouse and nonsymptomatic/symptomatic samples are summarized in a relative abundance–prevalence matrix (Fig. 6). This matrix shows for each differentially abundant zOTU its relative abundance (%) and its prevalence (color shade). Generally, when considering the rhizosphere, differentially abundant zOTUs showed higher relative abundance in nonsymptomatic compared to symptomatic plants (Fig. 6A). In greenhouses 2 and 3, a member of the Comamonadaceae family (zOTU1) and a zOTU identified as *Aeromonas* sp. (zOTU18) were significantly more abundant in nonsymptomatic compared to symptomatic plants (4.6% vs. 2.5% and 2.1% vs. 0.4%, respectively, in greenhouse 2; 30.6% vs. 18.5% and 6.6% vs. 4.1%, respectively, in greenhouse 3). Similarly, in greenhouses 1 and 2, a *Sphingobium* sp. (zOTU2) and *Flavobacterium* sp. (zOTU11) showed significantly higher relative abundance in nonsymptomatic plants (6.1% vs. 3.4% and 6.0% vs. 1.9%, respectively, in greenhouse 1; 14.4% vs. 6.5% and 1.6% vs. 0.8%, respectively, in greenhouse 2). An unknown bacterium (zOTU15) was significantly more abundant in nonsymptomatic compared to symptomatic plants in greenhouses 1 and 3 (6.9% vs. 0.8% in greenhouse 1; 2.7% vs. 1.3% in greenhouse 3, respectively). On the contrary, two zOTUs (zOTU4 and zOTU10) were significantly more abundant in the symptomatic compared to the nonsymptomatic plants and were identified as *Pseudomonas* sp. and *Flavobacterium* sp., respectively. The zOTU identified as *Flavobacterium* sp. (zOTU10) was significantly more abundant in symptomatic compared to nonsymptomatic plants in all three greenhouses (16.1% vs. 1.1% in greenhouse 1; 1.9% vs. 0.1% in greenhouse 2; and 0.4% vs. 0.0% in greenhouse 3). Likewise, the *Pseudomonas* sp. (zOTU4) showed significantly higher relative abundance in symptomatic plants from greenhouses 1 and 2 compared to nonsymptomatic plants (1% vs. 0.6% in greenhouse 1; 19.9% vs. 8.6% in greenhouse 2). BLAST analysis revealed that this *Pseudomonas* species most likely belongs to the *Pseudomonas fluorescens* or *Pseudomonas putida* group.

Interestingly, most zOTUs differentially abundant in the rhizosphere were also found to be differentially abundant in the endosphere (Fig. 6A and B). With regard to the endosphere, the zOTU belonging to the Comamonadaceae family (zOTU1) was significantly more abundant in the nonsymptomatic compared to the symptomatic plants in both greenhouse 1 (5.0% vs. 1.5%, respectively) and greenhouse 3 (3.3% vs. 0.1%, respectively). Also zOTU2, identified as *Sphingobium* sp., showed a significantly higher relative abundance in nonsymptomatic compared to symptomatic plants in greenhouse 2 (6.9% vs. 1.8%, respectively), as well as an unknown bacterium (zOTU15) in greenhouse 3 (7.6% vs. 1.0%, respectively). On the contrary, the other differentially abundant zOTUs were significantly more abundant in the symptomatic compared to the nonsymptomatic plants. Two of them were identified as *Flavobacterium* sp. (zOTU10 and zOTU30), of which zOTU10 was differentially abundant in both greenhouse 1 (11.2% vs. 0.7%, respectively) and greenhouse 2 (7.1% vs. 0.3%, respectively), while zOTU30 was only differentially abundant in greenhouse 2 (20.1% vs. 1.4%, respectively). Interestingly, zOTU10 showed also significant higher relative abundance in the rhizosphere of the symptomatic plants in all three greenhouses. Also a *Duganella* sp. (zOTU19) was significantly more abundant in the symptomatic compared to nonsymptomatic plants in greenhouse 1 (15.4 vs. 0.5%, respectively).

## Discussion

Although microbiome research has gained considerable attention in recent years, studies that examine the root microbiome of hydroponically grown crops are still limited, despite its importance in modern agriculture. Specifically, there is limited knowledge regarding the impact of pathogens on the microbial community in hydroponic systems. To fill this research gap, we conducted a study on the interaction between hydroponically grown lettuce and *P. cryptogea*. Our objective was to investigate how *P. cryptogea* influences the bacterial community in the rhizosphere and endosphere of lettuce plants grown in hydroponic systems. We sampled both symptomatic and nonsymptomatic plants from different greenhouses to ensure a comprehensive representation of the real-world scenario. However, it is important to note that, although plants without typical symptoms of *P. cryptogea* were considered nonsymptomatic plants, they could have been infected just before sampling. This is supported by the low levels of *P. cryptogea* DNA detected in most of the nonsymptomatic plants. We noticed that nonsymptomatic plants were predominantly located at the beginning of the gutters, while symptomatic plants were more commonly found towards the end. It is highly probable that a disparity in oxygen concentration along the gutters exist, with higher levels at the beginning and lower levels toward the end. Higher oxygen levels have a beneficial impact on plant growth, but can also influence bacterial communities and pathogen development (Chérif et al. 1997, Suyantohadi et al. 2010, Martínez-Arias et al. 2022).

Although microbial communities in the endosphere have long been overlooked regarding their role in plant health, endophytes have recently received increasing attention because of their intimate interaction with plants (Compant et al. 2019, 2021). Our results show that *P. cryptogea* can reach high densities in the endosphere following root infection. Nevertheless, its impact on the microbial community was less pronounced in the endosphere compared to the rhizosphere. With regard to alpha diversity, no significant differences were found between the symptomatic and nonsymptomatic plants for the endosphere samples, although it was shown earlier that the endophytic microbiome can be affected by pathogen infection (Proença et al. 2017, Suhaimi et al. 2017, Kaushal et al. 2020). For example, a lower OTU richness and diversity have been detected in the endosphere of healthy banana plants compared to those infected with *F. oxysporum* f. sp. *cubense* in the same field (Kaushal et al. 2020). However, it is important to highlight that in our study the rarefaction curves for the endosphere samples did not reach saturation, suggesting that alpha diversity may be underestimated and/or the impact of the pathogen might not be fully captured. This was particularly caused by the lack of specificity of the 16S rRNA gene primers, leading to an overamplification of mitochondrial and chloroplast plant DNA. Likewise, it was not possible to determine the bacterial densities in the endosphere samples by qPCR due to the nonspecificity of the primers. This can possibly be circumvented by using more specific primers or by adding peptide–nucleic acid PCR clamps, although the efficacy of both methods depends on the plant species (Lundberg et al. 2013, Mori et al. 2014, Thijs et al. 2017, Fitzpatrick et al. 2018). Regarding the rhizosphere samples, an increased alpha diversity was found for samples of symptomatic compared to nonsymptomatic plants, indicated by a significantly higher number of bacterial zOTUs in samples from *P. cryptogea* infected plants. Although we did not check for differences in root exudate composition and/or fatty acids of healthy and diseased plants in this study, it is highly probable that infection by *P. cryptogea* leads to a significant alteration in the composition of root exudates. This phenomenon is well-documented in previous studies exploring pathogen-induced changes in root exudation. Consequently, these altered root exudates can act as signals and attract plant-associated microorganisms in an attempt to overcome the infection, also known as the so-called “cry-for-help” strategy (Lombardi et al. 2018, Yuan et al. 2018, Rolfe et al. 2019). Accordingly, several studies have shown that pathogen invasion increases microbial diversity in the rhizosphere. For example, the rhizosphere of hydroponically grown tomato plants infected with rhizogenic agrobacteria exhibited higher zOTU richness compared to their healthy counterparts (Vargas et al. 2022). Also a higher diversity of Gammaproteobacteria was found in the rhizosphere and phyllosphere of pot-cultivated lettuce plants infested by *R. solani* compared to healthy lettuce plants (Erlacher et al. 2014). However, several studies have also reported the opposite phenomenon, wherein there is a decrease in microbial diversity in the rhizosphere following invasion of a pathogen (Trivedi et al. 2012, Zhang et al. 2017, Wei et al. 2018).

In terms of beta diversity, significant differences were found between samples of nonsymptomatic and symptomatic plants for each greenhouse. Similarly, Vargas et al. (2022) observed significant differences in bacterial communities between healthy and rhizogenic agrobacterium-infected tomato plants in hydroponics. In soil-bound cultivation, a distinct gammaproteobacterial community was found in nonsymptomatic lettuce plants compared to plants infected by *R. solani* (Erlacher et al. 2014). Infestation of *R. solanacearum* in soil-grown tomato plants has also led to changes in the rhizosphere microbiome composition (Wei et al. 2018). Also upon artificial inoculation of pathogens, changes in the microbiome have been observed. For example, infection with *Verticillium dahliae* clearly changed the tomato root microbiome compared to mock-inoculated tomato plants (Snelders et al. 2020).

Apart from differences between symptomatic and nonsymptomatic plants, major significant differences in bacterial communities were also found between greenhouses for both the rhizosphere and endosphere samples. Considering the variability in cultivation practices, differences in beta diversity were not unexpected. For instance, different lettuce varieties (butterhead lettuce vs. multicolor lettuce) and different cultivars were grown. Previous research suggests that cultivars may have a significant impact on microbial communities in the rhizosphere, as evidenced for example in *Brassica napus*, where two different cultivars showed remarkable differences in the endophytic bacterial populations and total microbial load, even in the seed stage (Granér et al. 2003). Although the substrate, fertigation and climatic conditions were the same among the greenhouses, differences in the use of plant protection products, water disinfection strategies, or environmental conditions might have affected the composition of the microbial root community as well, as demonstrated in previous studies (Vallance et al. 2011, Sangiorgio et al. 2022). For example, it was shown that the use of Teldor WG50^®^ (active ingredient fenhexamid) against *Botrytis cinerea* in the nutrient solution of an hydroponic tomato cultivation system had an influence on the microbial community composition (Alsanius et al. 2013). While no information is available on the use of plant protection products in greenhouses 2 and 3, chemical pesticides against downy mildew were sprayed in greenhouse 1. Also, the microbial load and/or microbial composition of the nutrient solution may have been different, as samples were taken at different moments during the growing season (June and August, 2021) and each greenhouse utilized rainwater from their greenhouse basin for the nutrient solution.

Despite the variability among greenhouses, a differential abundance analysis revealed several zOTUs, which were differentially abundant between the rhizosphere of nonsymptomatic and symptomatic plants in at least two out of three greenhouses. These included members of *Pseudomonas* and *Flavobacterium*, which were particularly associated with symptomatic plants. Although certain *Pseudomonas* spp. could be pathogenic for plants, BLAST analysis of the zOTU associated with symptomatic plants identified as *Pseudomonas* sp. showed that it most likely belongs to the *P. fluorescens* or *P. putida* group. These *Pseudomonas* spp. are well-known for their biocontrol potential and are often referred to as plant growth-promoting rhizobacteria (PGPR). They can exhibit both direct and indirect effects to promote plant health. As direct effect, *Pseudomonas* spp. can produce bacterial allelochemicals, such as antibiotics and siderophores, while indirectly they can promote plant health through IR. The production of bacterial allelochemicals by *Pseudomonas* spp. and other PGPR can inhibit the growth of plant pathogens and provide a competitive advantage to the plant (Santoyo et al. 2012, Dorjey et al. 2017). Furthermore, the induction of systemic resistance by PGPR can activate the plant’s defense mechanisms and provide long-term protection against pathogen attacks (Choudhary et al. 2007). For instance, *Pseudomonas* strains isolated from both the phyllosphere and rhizosphere of potato plants have been found to exhibit antagonistic potential against *Phytophthora infestans* and other potato pathogens when cocultivated in the lab (Guyer et al. 2015, Hunziker et al. 2015). These *Pseudomonas* strains produce volatiles such as 1-undecene, which inhibit mycelial growth, as well as zoospore germination and release (Hunziker et al. 2015). Also, it was shown that some *Pseudomonas corrugata* strains exhibit biocontrol activity against *Phytophthora* blight of pepper (caused by *Phytophthora capsici*) through successful colonization of plant roots (Sang and Kim 2014). BLAST analysis of the zOTUs identified as *Flavobacterium* sp. was less conclusive about the species identity and consequently their characteristics. However, to our knowledge, no *Flavobacterium* spp. with plant pathogenic characteristics are known, while their plant-beneficial effects (e.g. plant growth-promoting characteristics and antimicrobial activity) are well-documented (Alexander and Stewart 2001, Sang et al. 2008, Soltani et al. 2010, Kolton et al. 2014, Kwak et al. 2018, Carrión et al. 2019). The endophytic volatile-producing strain *Flavobacterium johnsoniae* GSE09, isolated and characterized from surface-sterilized roots of pepper plants, has demonstrated the ability to colonize pepper roots as well as the rhizosphere, resulting in reduced colonization by *Phytophthora capsici*, and consequently a diminished disease severity in pepper plants (Sang et al. 2008, Sang and Kim 2012). Moreover, it was found that *Flavobacterium* spp. are often highly abundant in the plant rhizosphere, where they can colonize plant roots and have a role in increasing the plant immune response (Kolton et al. 2014). These characteristics make *Pseudomonas* and *Flavobacterium* spp. promising candidates for biocontrol strategies in agriculture, including biocontrol of *P. cryptogea*. Interestingly, we found that these bacteria were particularly associated with *P. cryptogea*-infected plants, in line with the “cry-for-help” hypothesis (Bakker et al. 2018, Rolfe et al. 2019, Rizaludin et al. 2021). Therefore, given their known biocontrol potential and natural occurrence in the hydroponic cultivation of lettuce, we hypothesize that the *Pseudomonas* sp. and *Flavobacterium* spp. identified in this study could be explored as biocontrol organism (BCO) against *P. cryptogea*. Previous studies have demonstrated that both rhizosphere microorganisms and endophytes can restrict infection by *Phytophthora* spp. This illustrates the power of isolating plant beneficial microbes from the plant-associated microbiome as a potential source of BCOs (Arnold et al. 2003, Abraham et al. 2013, Acebo-Guerrero et al. 2015, Islam et al. 2016, de Vries et al. 2018, Xi et al. 2022). For instance, pretreatment of cacao tree (*Theobroma cacao*) roots with *Pseudomonas chlororaphis*, which was isolated from the rhizosphere of the tree, was found to reduce symptom severity following *Phytophthora palmivora* inoculation (Acebo-Guerrero et al. 2015). Moreover, inoculation of cacao leaves with fungal endophytes, isolated from healthy cacao trees, showed a significant decrease in leaf mortality and necrosis after inoculation with *Phytophthora* sp. (Arnold et al. 2003). However, further research is needed to explore the potential of the identified species as BCO against *P. cryptogea* infection in hydroponically grown lettuce crops.

Altogether, our study has clearly shown that the bacterial community composition of hydroponically grown lettuce is largely dependent on the investigated greenhouse. Further, our study indicates that *P. cryptogea* infection has a strong impact on the composition of the bacterial community in the rhizosphere and endosphere. Differential abundance analysis revealed *Pseudomonas* spp. and *Flavobacterium* spp. as potentially important genera associated with symptomatic plants. Further research is needed to further explore their potential as BCOs.

## Supplementary Material

fiae010_Supplemental_Files
