# Supplementary material for: Unveiling the microbiome of hydroponically cultivated lettuce: impact of Phytophthora cryptogea infection on plant-associated microorganisms
Source: FEMS Microbiol Ecol. 2024 Feb 5;100(3):fiae010. doi: 10.1093/femsec/fiae010 (PMC10872686; doi:10.1093/femsec/fiae010)
Supplement: fiae010_Supplemental_Files [file fiae010_supplemental_files.zip › Vlasselaer_Supporting_data Information_(Figures).docx]

# Supporting Information


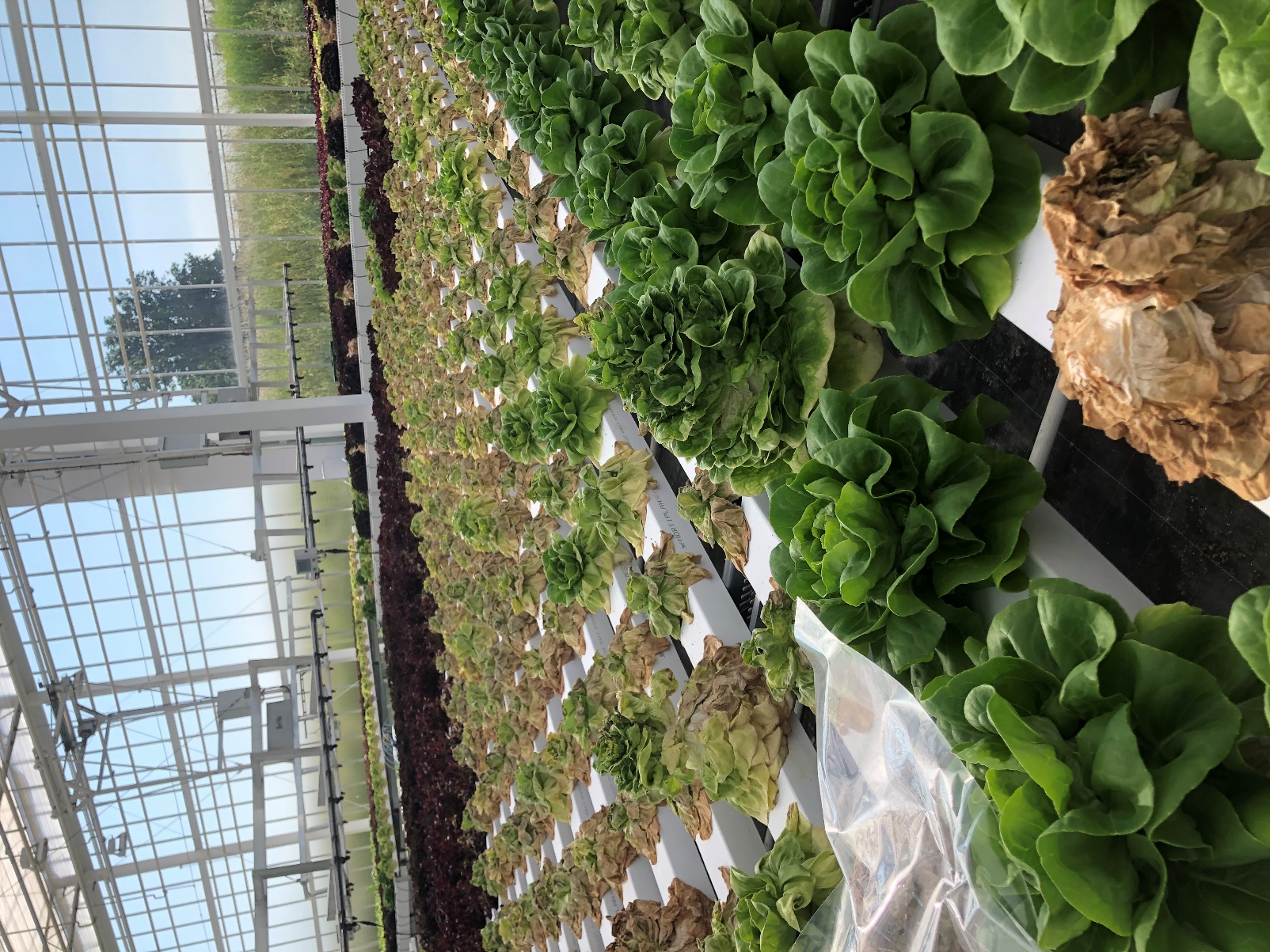


Figure S1: Hydroponic lettuce invaded by the oomycete Phytophthora cryptogea. The roots were first infected, after which the entire crop will show rotting symptoms.


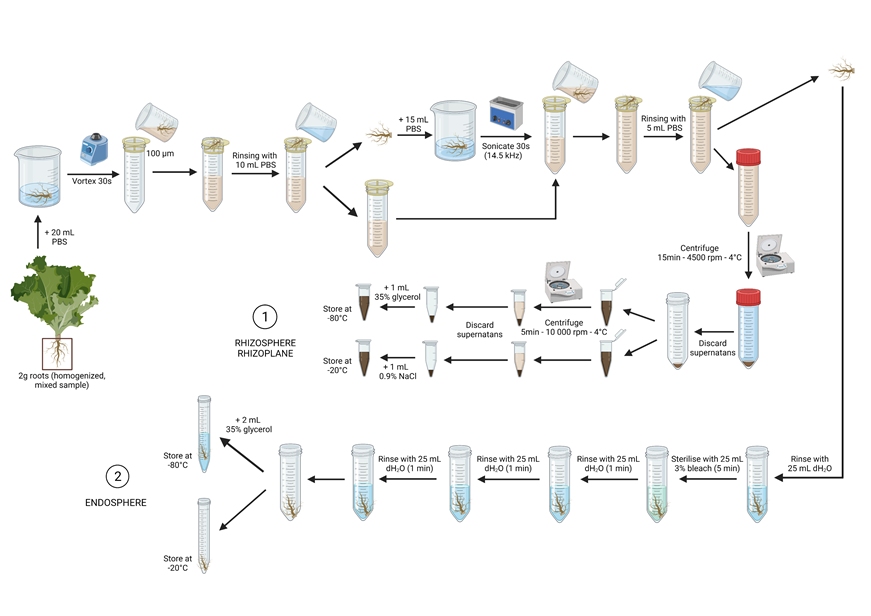


Figure S2: Schematic representation of how lettuce root samples were processed for sampling rhizosphere and endosphere microbial communities.


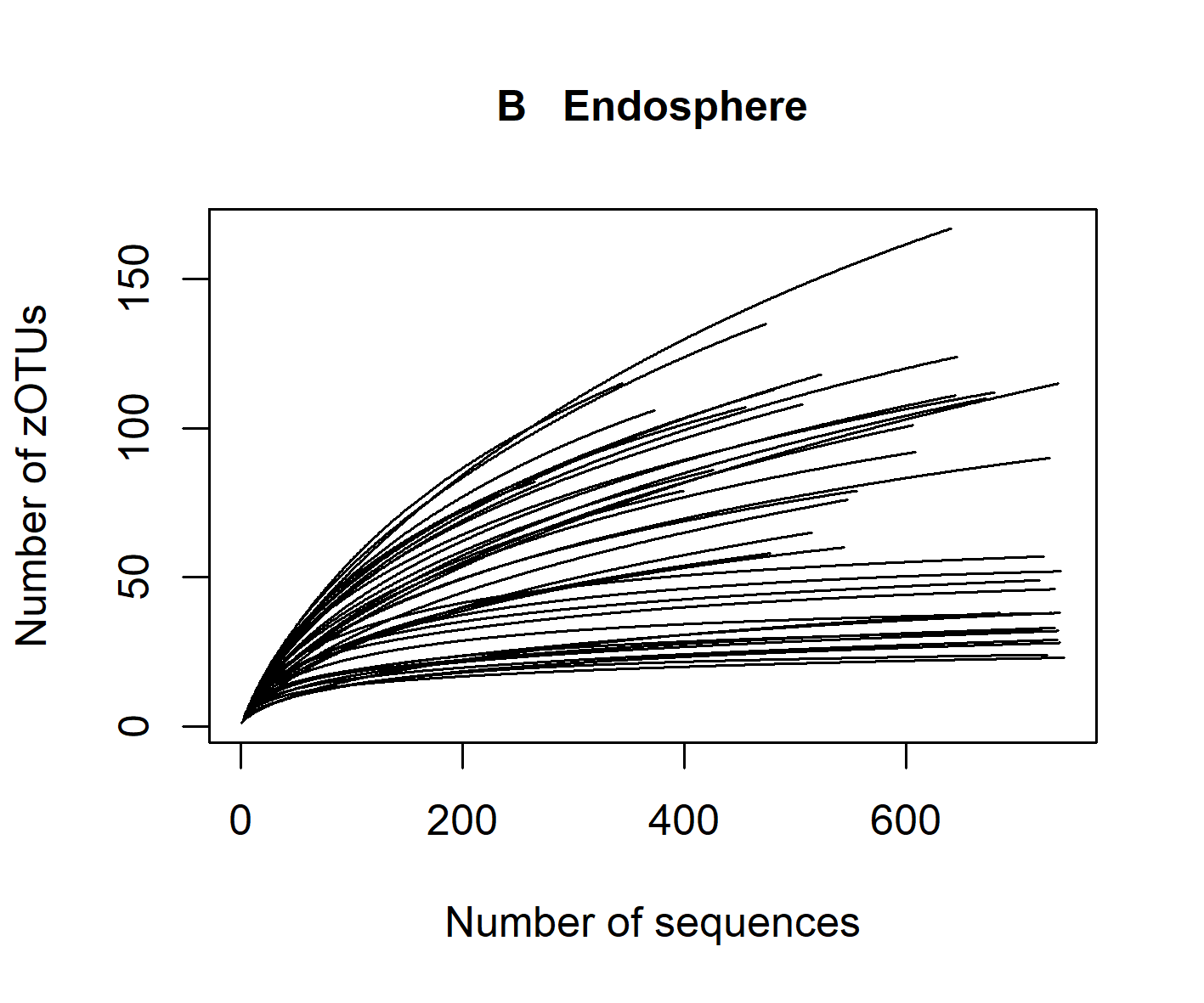

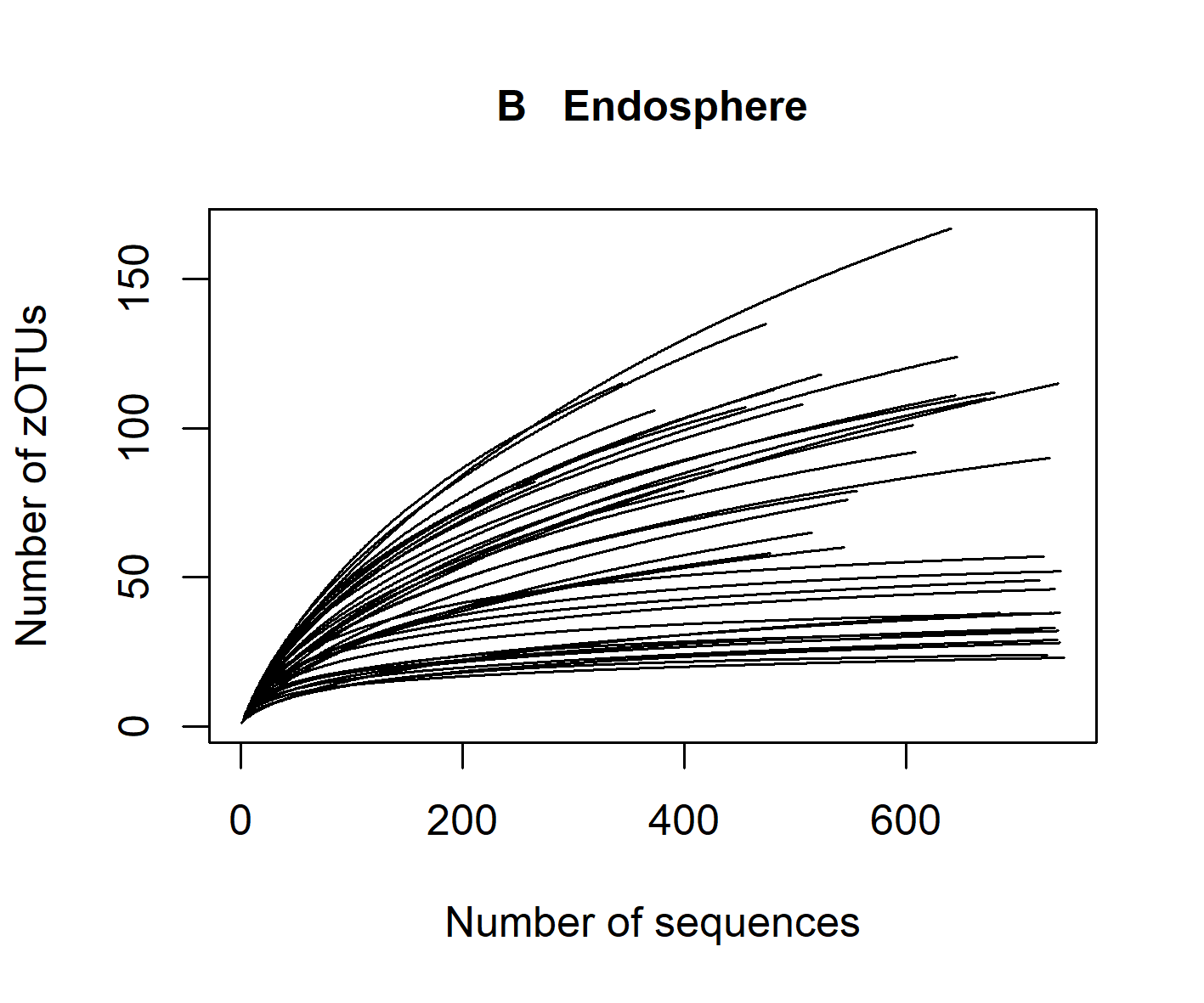

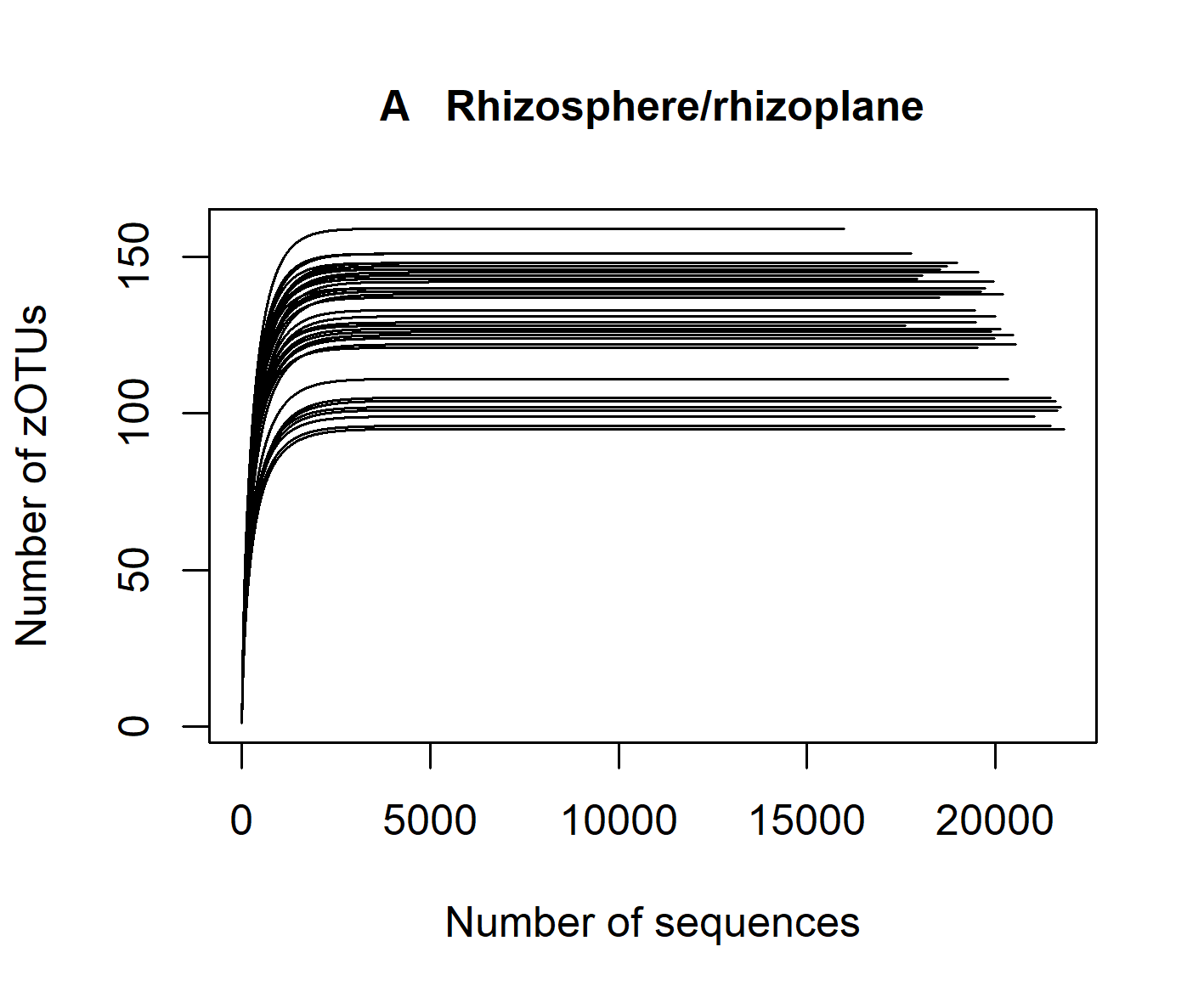

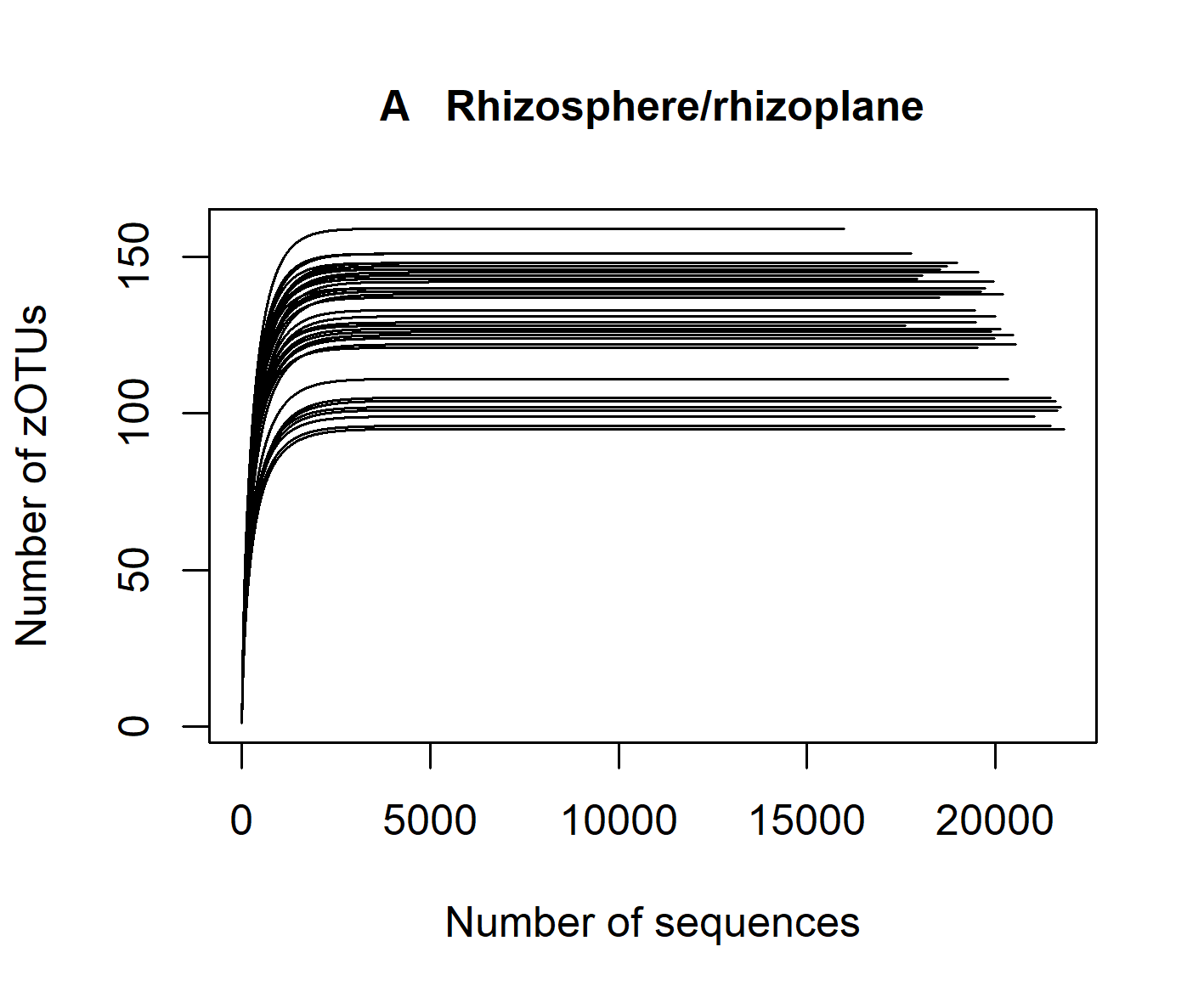


Figure S3: Rarefaction curves for rhizosphere (A) and endosphere (B) samples. Rarefaction curves for rhizosphere samples approached saturation, while those for the endosphere tended to approach saturation.


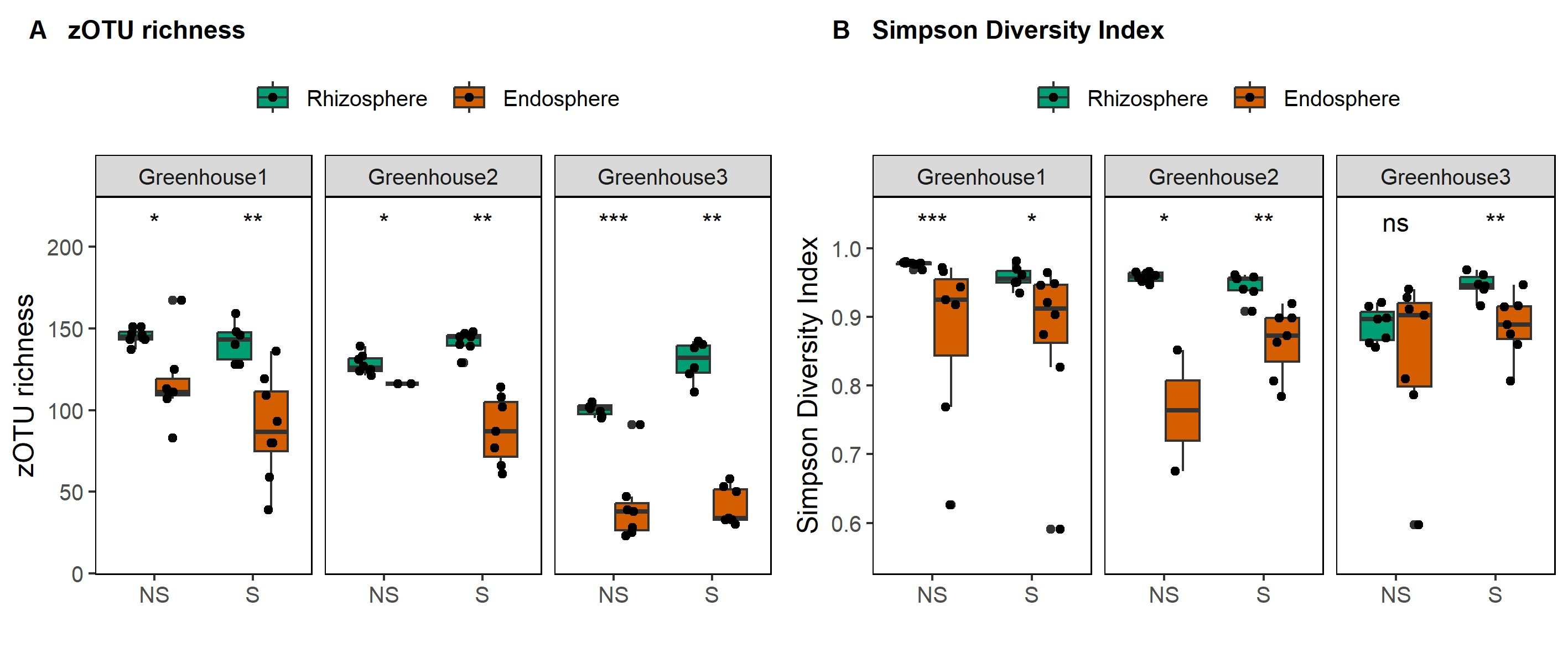
Figure S4: Zero-radius Operational Taxonomic Unit (zOTU) richness (A) and Simpson Diversity Index (B) of bacterial communities of root rhizosphere and endosphere collected at three different hydroponic lettuce growers for both symptomatic and non-symptomatic plants. The lower, middle and upper lines of the boxplots correspond to the first quartile, median and third quartile, respectively, while the whiskers represent the range from the minimum to the maximum. Data points represent the different replicates. Significant differences between endosphere and rhizosphere are shown by an asterisk (p > 0.05 (ns), p ≤ 0.05 (*), p ≤ 0.01 (**) and p ≤ 0.001 (***)).


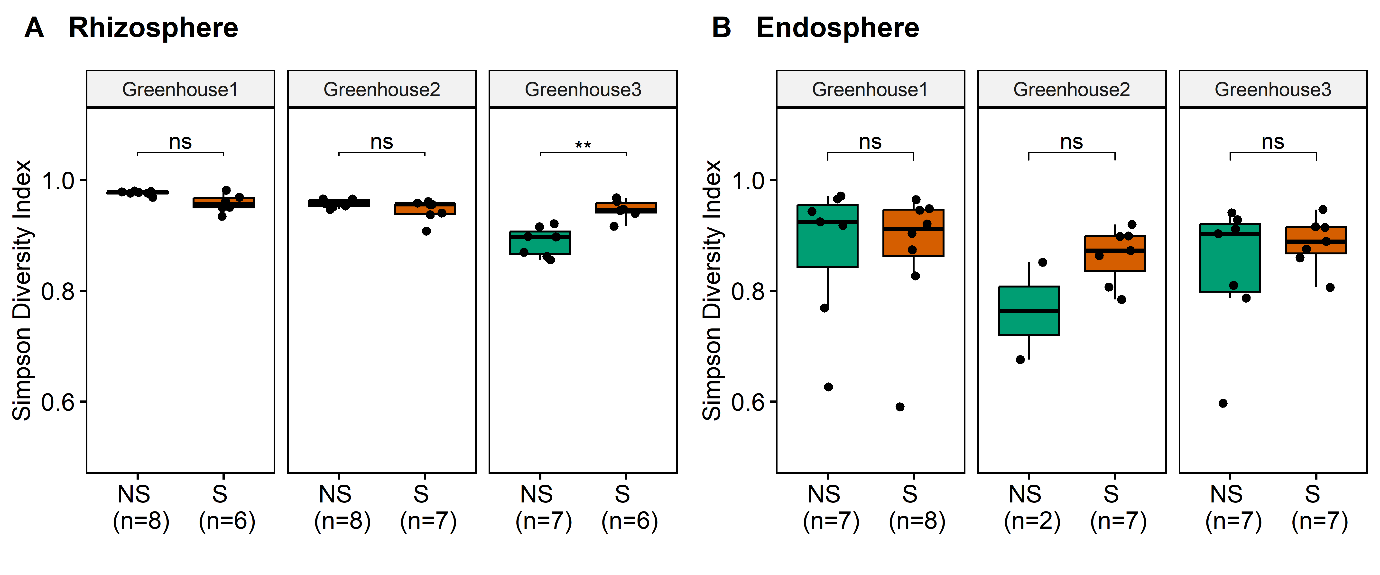


Figure S5: Simpson Diversity Index of bacterial communities of the rhizosphere (A) and root endosphere (B) of lettuce plants collected at three hydroponic lettuce greenhouses for both non-symptomatic (NS) and symptomatic (S) plants. The lower, middle and upper lines of the boxplots correspond to the first quartile, median and third quartile, respectively, while the whiskers represent the range from the minimum to the maximum. Data points represent the different replicates (number provided between brackets). Significant differences between symptomatic and non-symptomatic plants are indicated by one or more asterisks (p > 0.05 (ns), p ≤ 0.05 (*) and p ≤ 0.01 (**)).


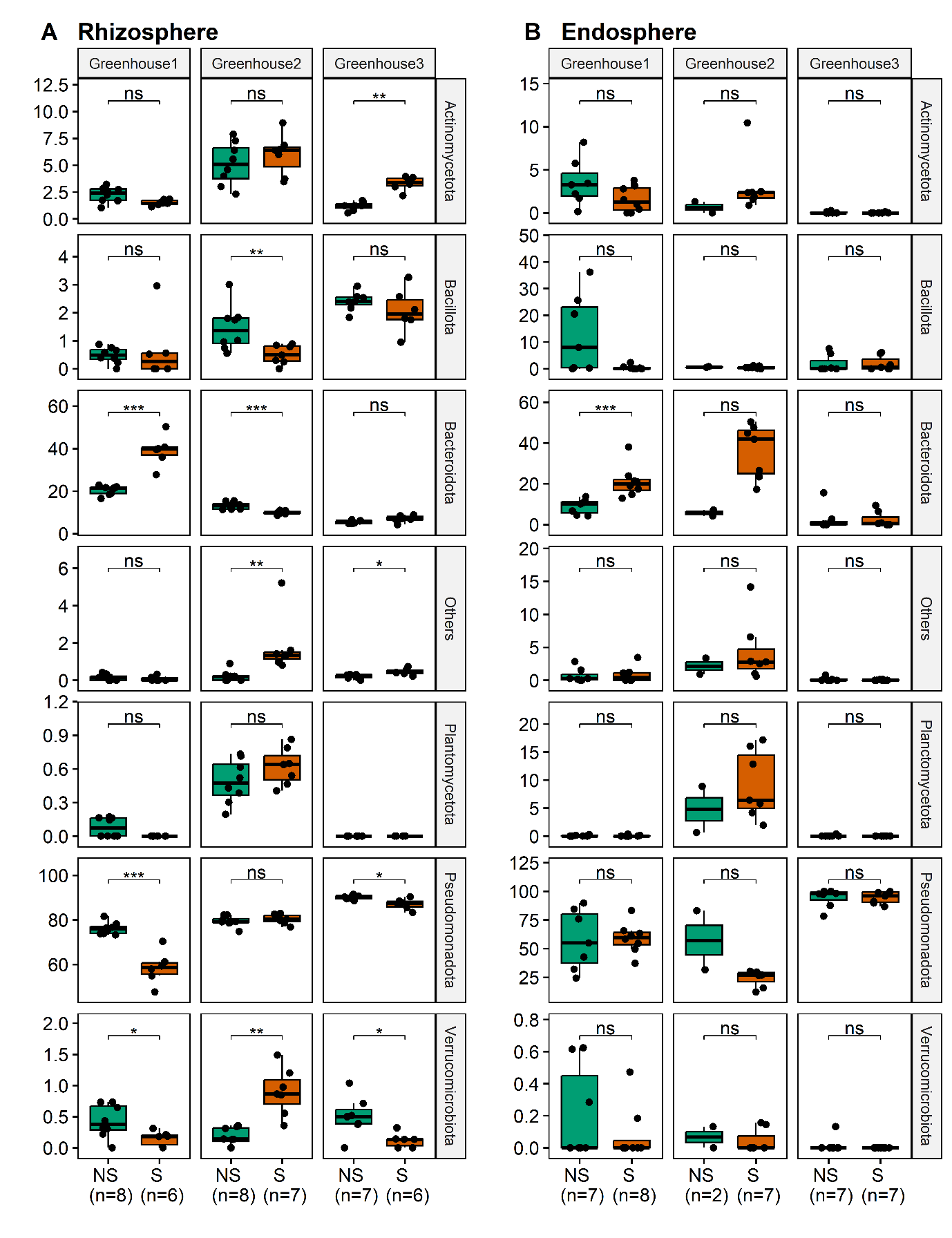


Figure S6: Relative abundance for the most abundant phyla in rhizosphere and endosphere samples of both non-symptomatic and symptomatic plants. The lower, middle and upper lines of the boxplots correspond to the first quartile, median and third quartile, respectively, while the whiskers represent the range from the minimum to the maximum. Data points represent the different replicates (number provided between brackets). Significant differences between endosphere and rhizosphere are shown by an asterisk (p > 0.05 (ns), p ≤ 0.05 (*), p ≤ 0.01 (**) and p ≤ 0.001 (***)).


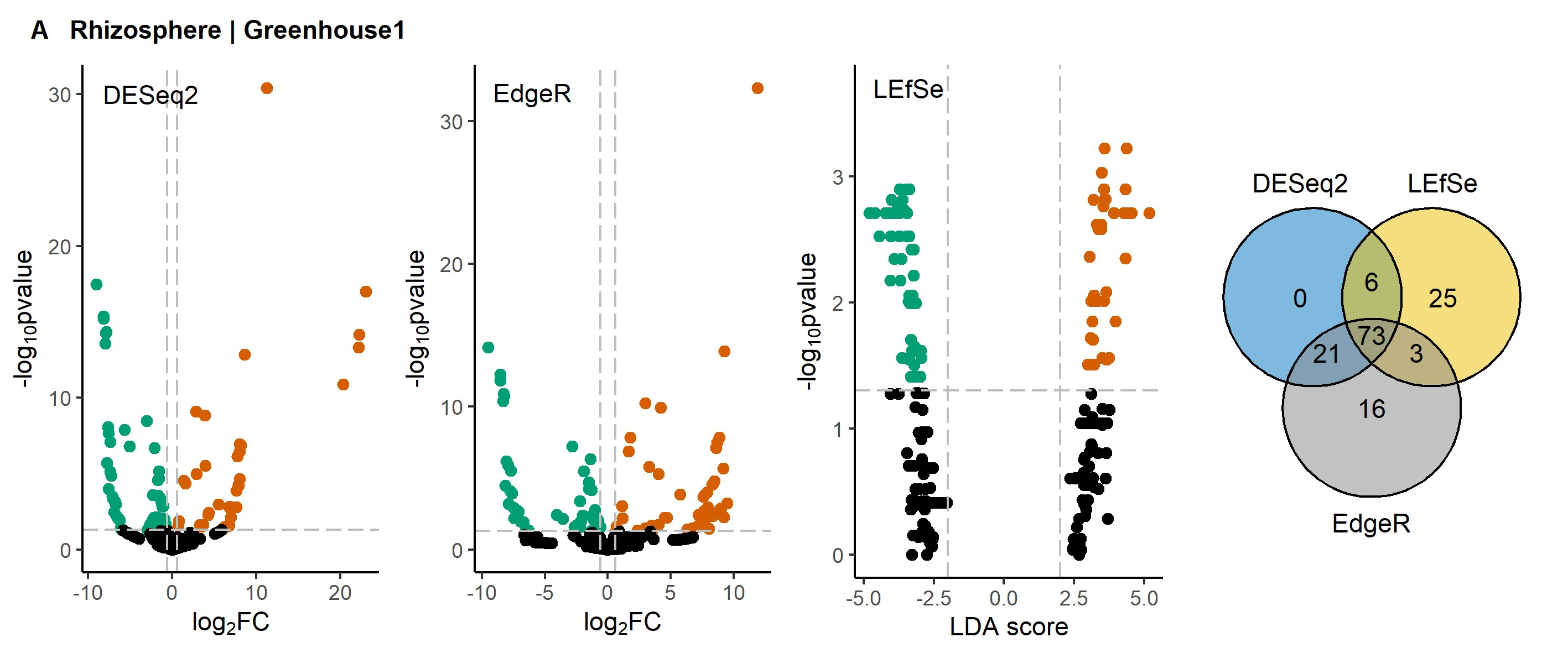


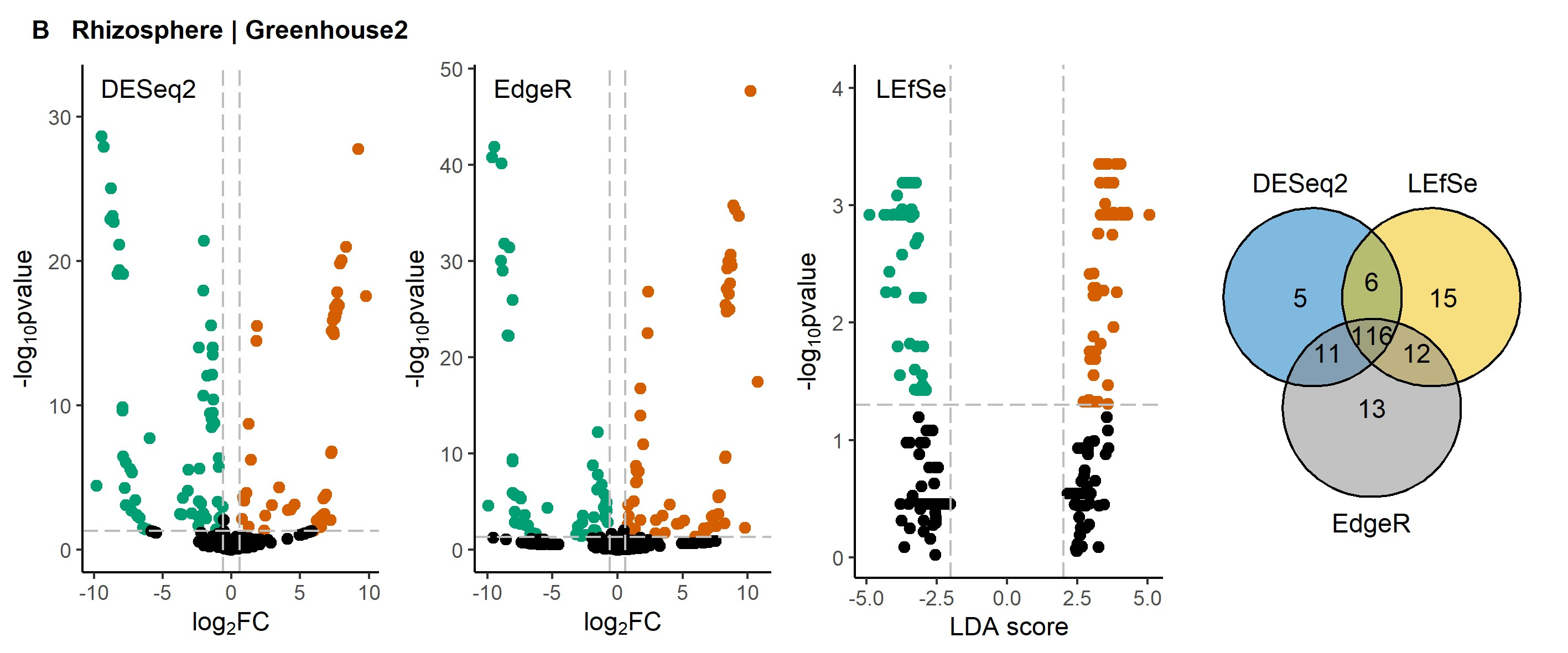


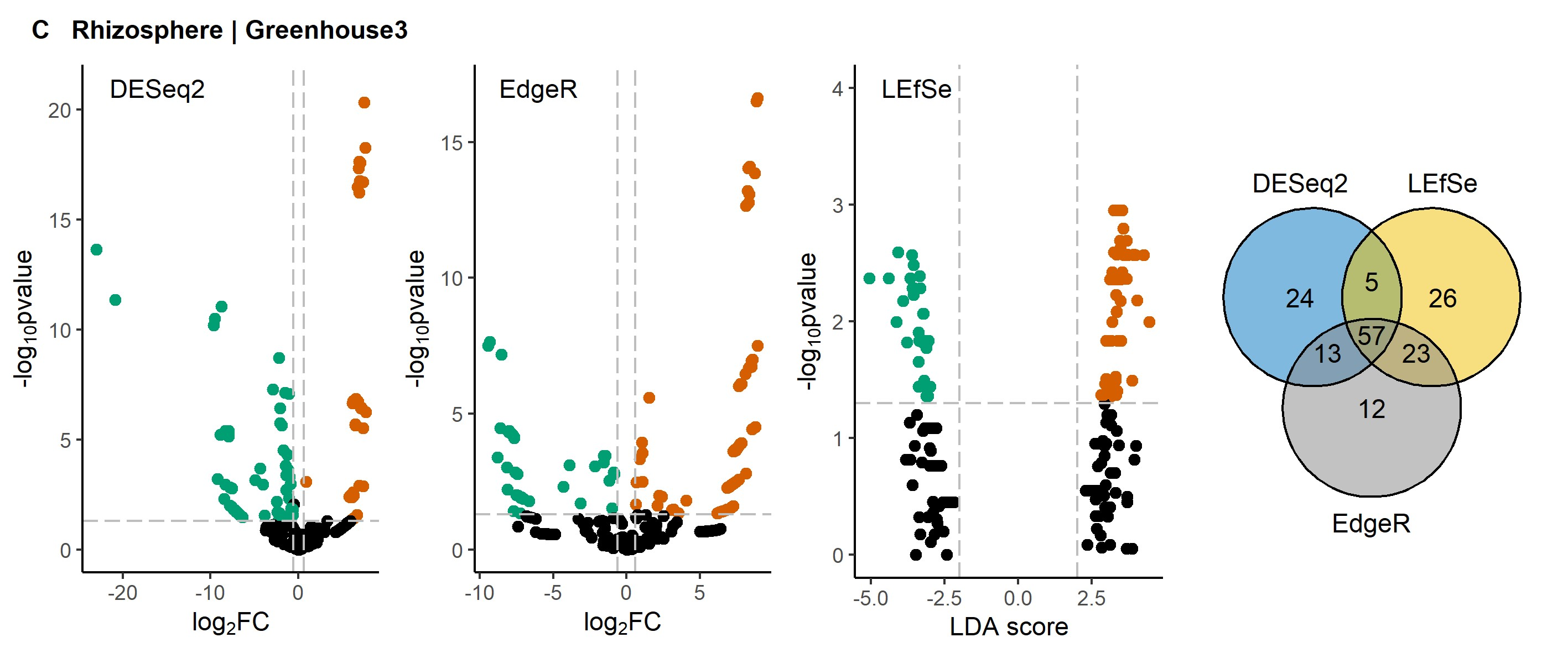


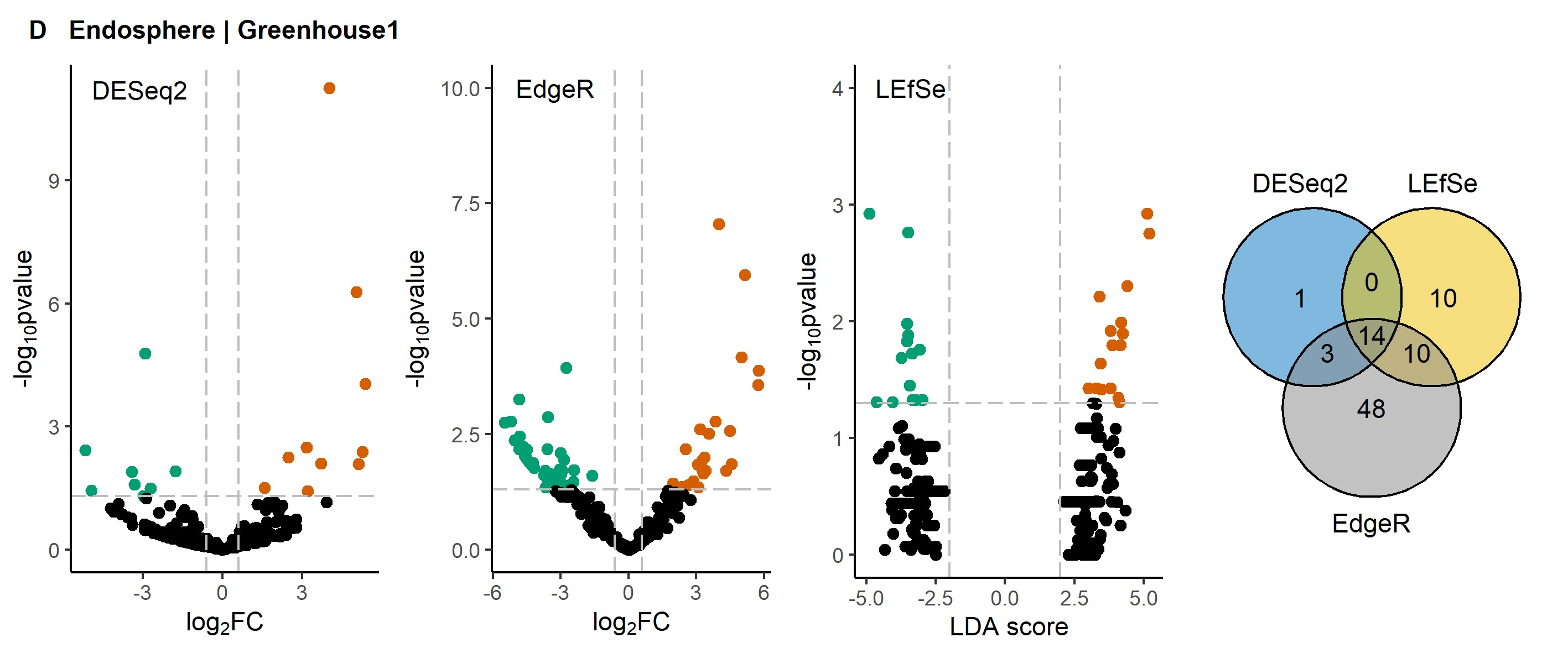


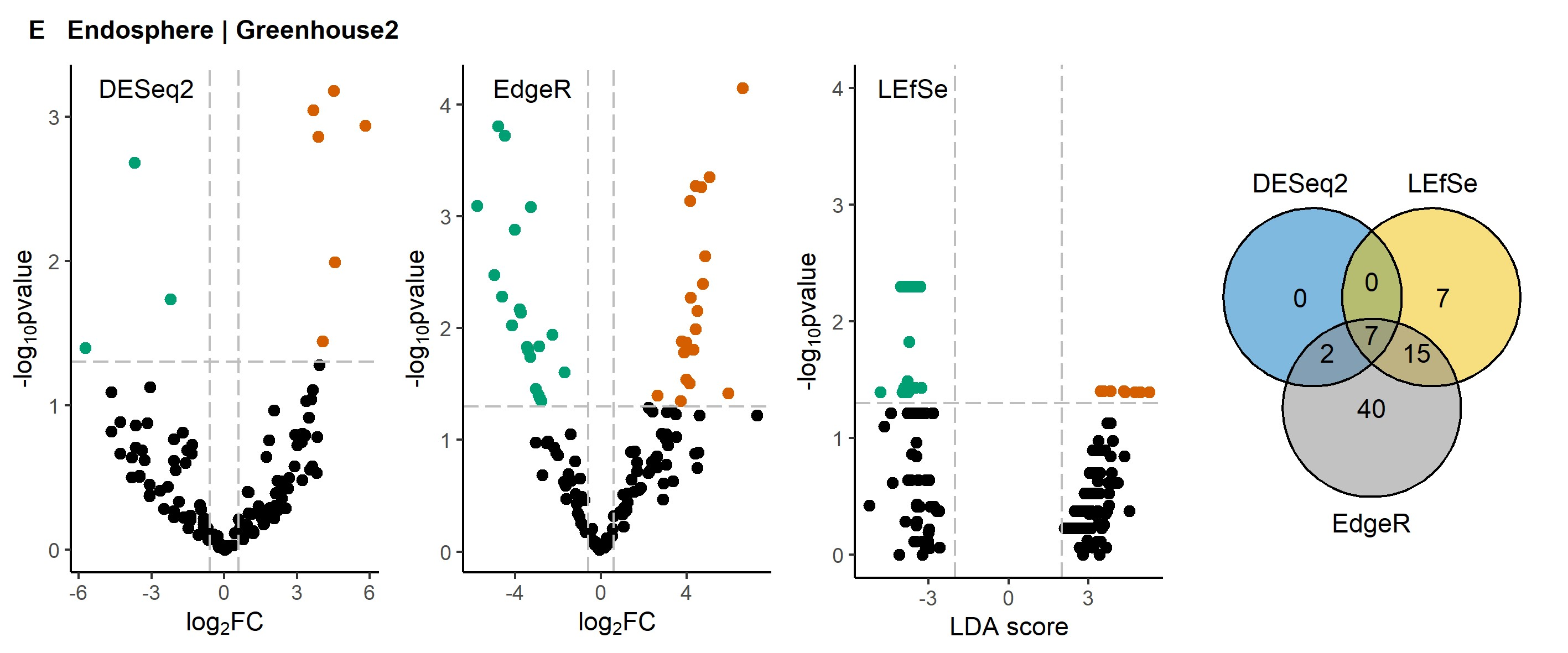


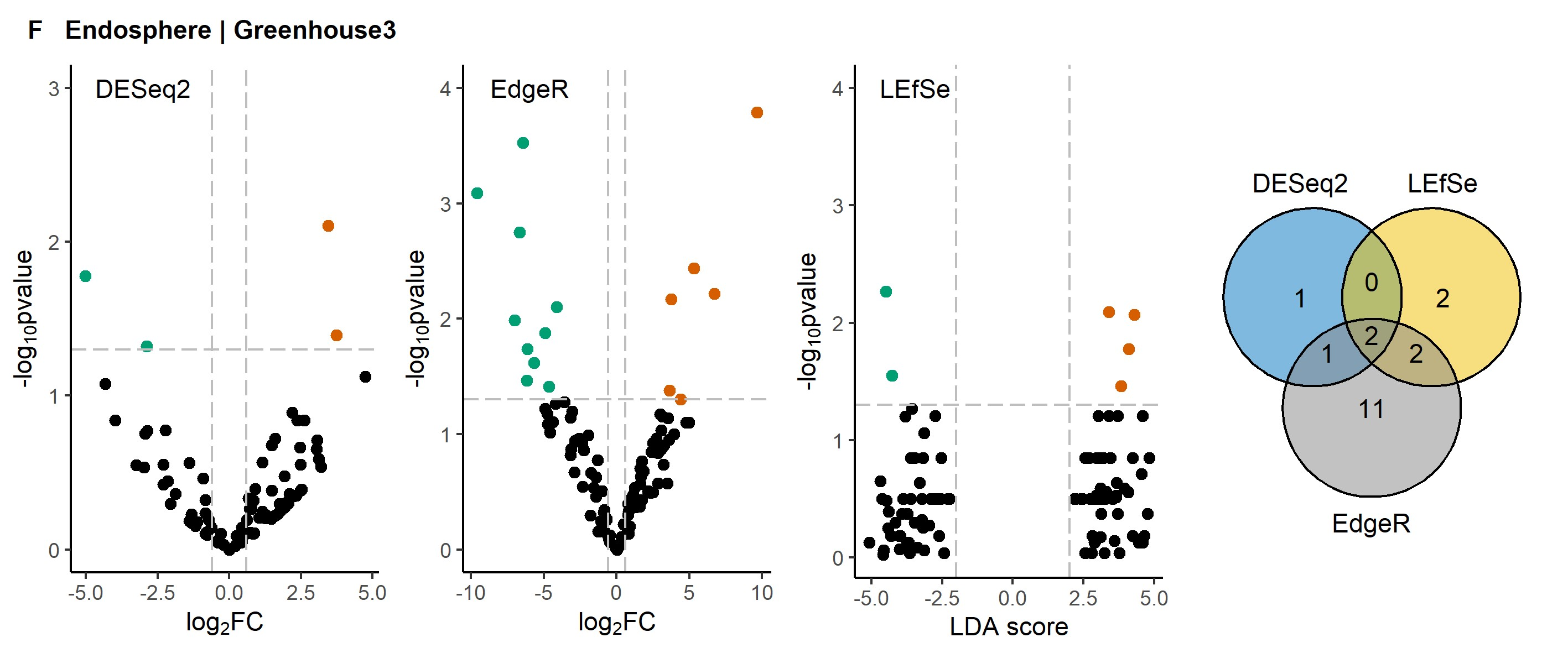


Figure S7: Volcano plots for the DESeq, EdgeR and LEfSe analysis , respectively, and a Venn diagram showing the unique and shared zero Operational Taxonomic Units (zOTUs) for the different analysis methods for the rhizosphere samples of grower 1 (A), grower 2 (B) and grower 3 (C) and for the endosphere samples of grower 1 (D), grower 2 (E) and grower 3 (F), respectively. Orange dots are representing the zOTUs that are more abundant in symptomatic (S) samples compared to non-symptomatic (NS) samples, while green dots are representing zOTUs that are more abundant in non-symptomatic (NS) samples compared to symptomatic (S) samples. Black dots represent the zOTUs that are not differentially abundant in symptomatic (S) compared to non-symptomatic (NS) plants.


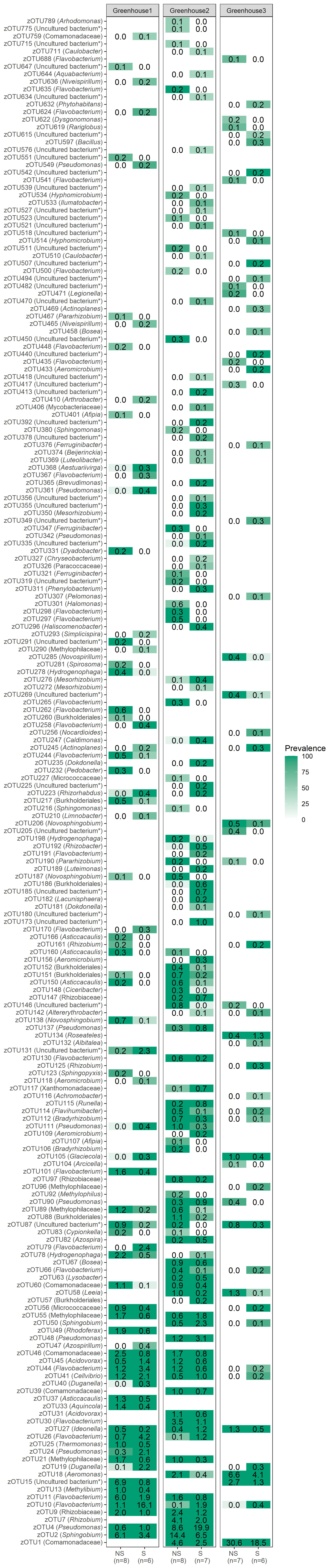


Figure S8: Summary of the relative abundance and prevalence of the zero-radius Operational Taxonomic Units (zOTUs) which are differentially abundant between non-symptomatic (NS) and symptomatic (S) plants for the **rhizosphere** according to the combined results of the DESeq2, EdgeR and LEfSe analyses. For each zOTU, the relative abundance is given as a number (%), while the color represents prevalence (i.e. fraction of samples in which the zOTU was present; white is absent). The number of replicates included is given between brackets. zOTUs are identified by a BLAST search against type materials in GenBank. When no significant similarity was found with type materials (< 97%), the BLAST analysis was performed against the entire GenBank (indicated with an asterisk). Identifications were performed at genus level; when identical scores were obtained for different genera, identifications were performed at family level.


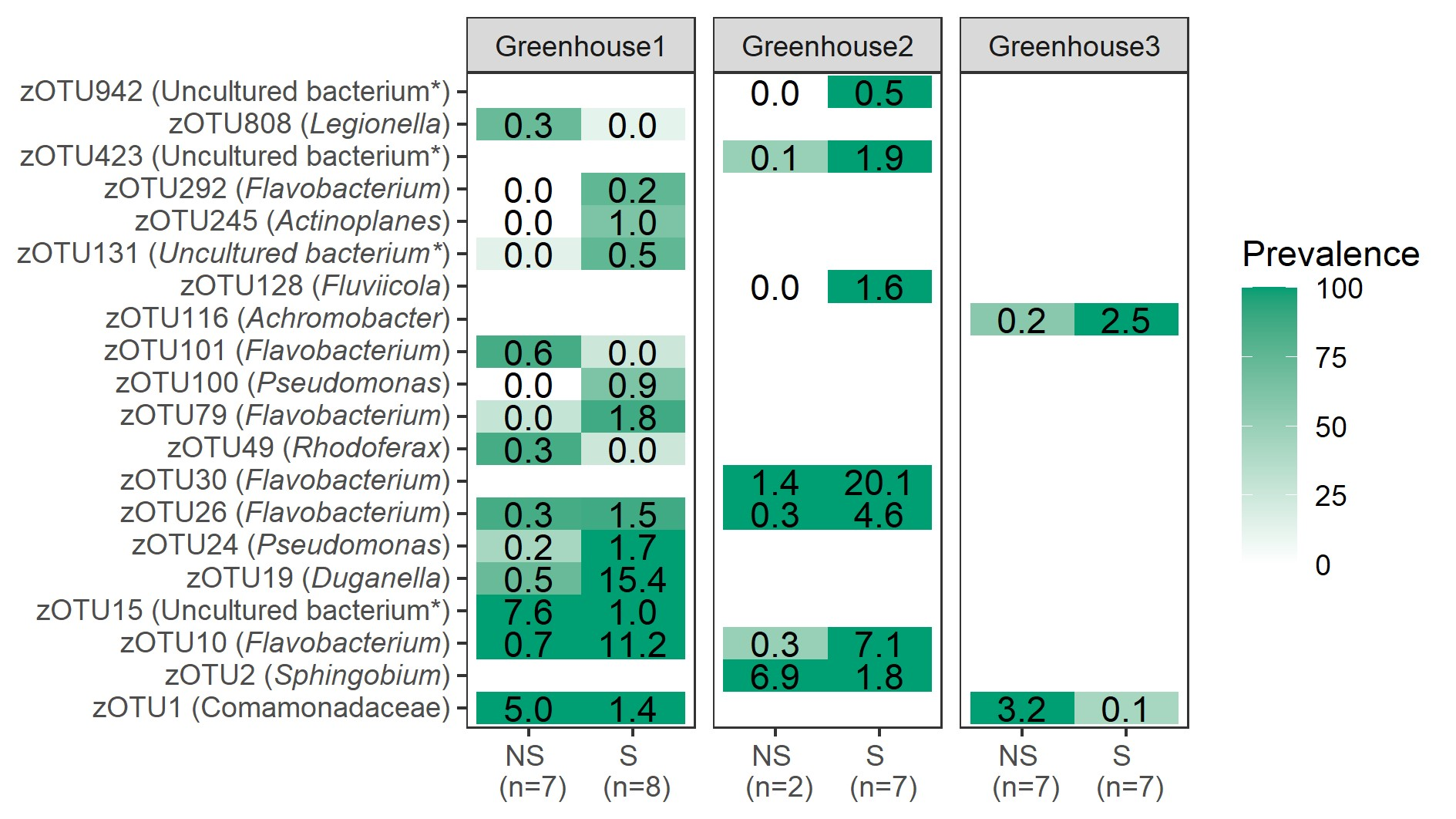


Figure S9: Summary of the relative abundance and prevalence of the zero-radius Operational Taxonomic Units (zOTUs) which are differentially abundant between non-symptomatic (NS) and symptomatic (S) plants for the **endosphere** according to the combined results of the DESeq2, EdgeR and LEfSe analyses. For each zOTU, the relative abundance is given as a number (%), while the color represents prevalence (i.e. fraction of samples in which the zOTU was present; white is absent). The number of replicates included is given between brackets. zOTUs are identified by a BLAST search against type materials in GenBank. When no significant similarity was found with type materials (< 97%), the BLAST analysis was performed against the entire GenBank (indicated with an asterisk). Identifications were performed at genus level; when identical scores were obtained for different genera, identifications were performed at family level.
